# Supplementary material for: Phenotypic characterization of single CD4+ T cells harboring genetically intact and inducible HIV genomes
Source: Nat Commun. 2023 Feb 27;14:1115. doi: 10.1038/s41467-023-36772-x (PMC9971253; doi:10.1038/s41467-023-36772-x)
Supplement: Supplementary file 1 — Supplementary Information [file 41467_2023_36772_MOESM1_ESM.pdf]

Supplementary Information files

Supplementary Fig. 1

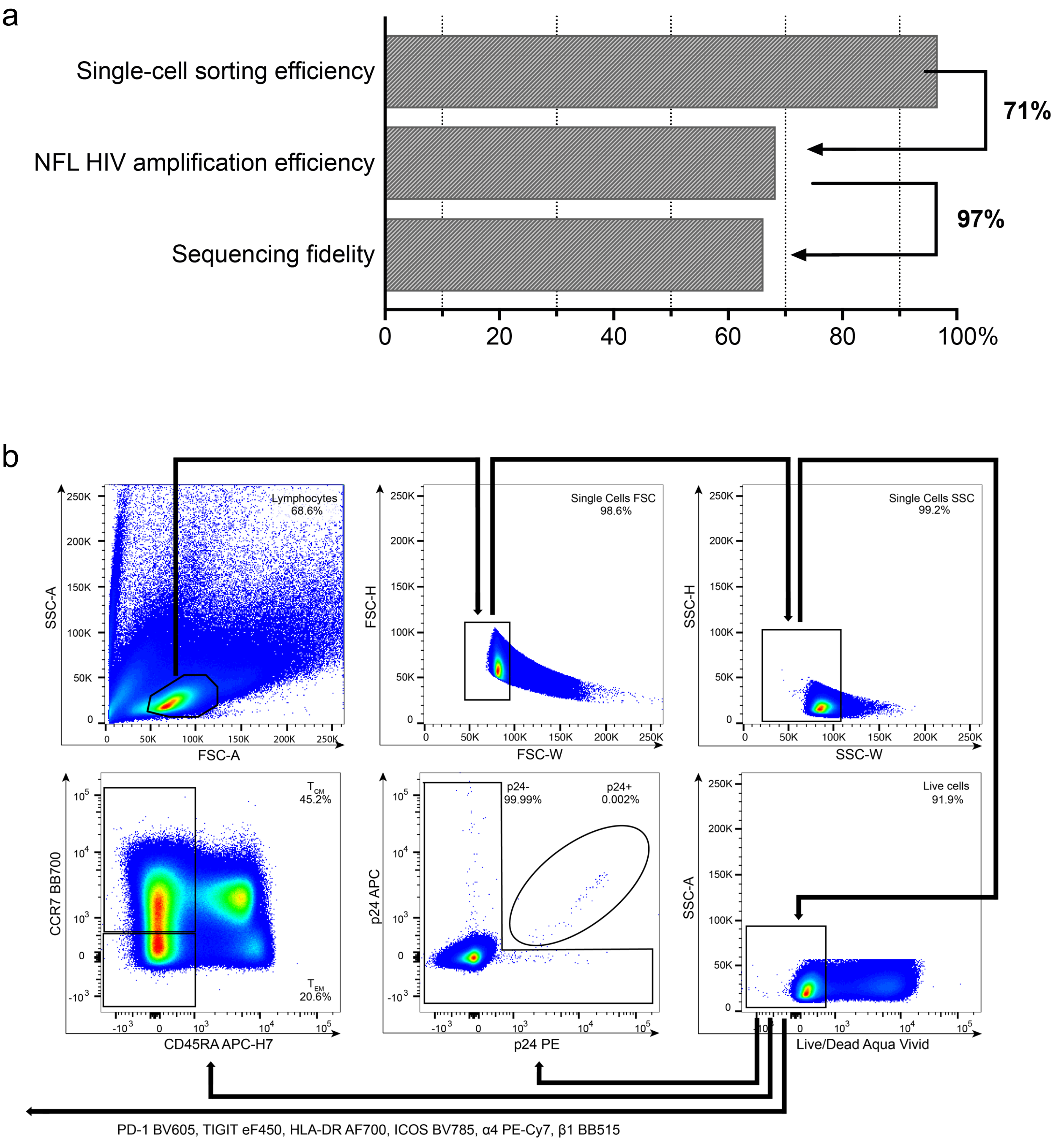

Supplementary Fig.1 Efficiency of the approach. a ACH-2 cells were single sorted and subjected to near full-length HIV genome amplification. Amplified products were barcoded and sent for PacBio next-generation sequencing. Single sorted ACH2 cells were retrieved from 96.7% of the wells (detectable CD3 gene by qPCR) and 70.1% of the genomes were successfully amplified. Proviral sequences from 93.0% of the amplified near full-length genomes were identical to each other and matched the MN691959.1 HIV-1 isolate ACH-2 reference (https://www.ncbi.nlm.nih.gov/nuccore/MN691959). The proportion of single-cell sorting efficiency, NFL HIV amplification efficiency, and sequencing fidelity are indicated. **b** Gating strategy for p24+ single-sorting cells, without indexing strategy. Dot plots from a representative participant are shown.

Supplementary Fig. 2

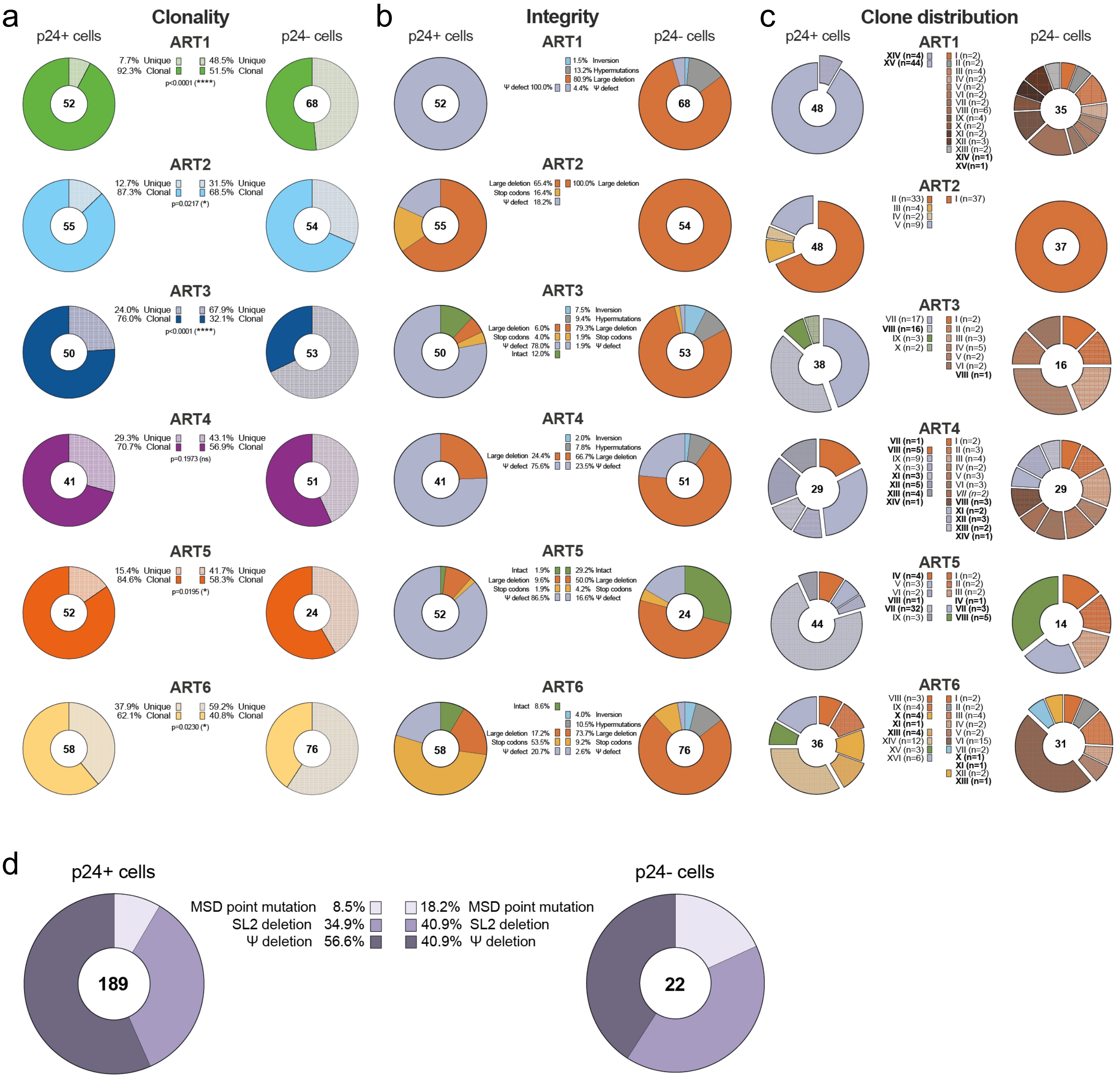

Supplementary Fig.2 Integrity and clonality of HIV genomes retrieved from p24+ and p24- cells for each participant. a Pie charts representing the proportions of unique (dotted color) and clonal (solid color) proviral sequences retrieved from p24+ and p24- cells. Percentages are indicated. The number of proviral sequences analyzed is indicated in each pie chart. For each participant, two-sided Fisher's exact test was used to determine differences in clonality between the two populations (*: $p < 0.05$; ****: $p < 0.0001$). **b** Pie charts representing the proportions of genomes with different types of genetic defects in p24+ and p24- cells. Genetic defects are color-coded and their percentages are indicated. The total number of proviral sequences analyzed is indicated in each pie chart. **c** Distribution and genetic integrity of the clonally expanded clones. Each fraction of the pie chart represents a clonal expansion and is color-coded according to the type of defect as in B. The number of individual cells belonging to each clone is indicated. The total number of clonally expanded proviral sequences analyzed is indicated in each pie chart. Clones in bold are shared between p24+ and p24- cells. **d** Proportions of Ψ defective proviruses with major splicing donor site point mutation (dark purple), stem loop 2 deletion (purple) and larger psi deletion (light purple) in p24+ and p24- cells. The number of proviral sequences analyzed is indicated in each pie chart. Differences in the contribution of each defect to the total population was performed using the two-sided Fisher's exact test.

Supplementary Fig. 3

a

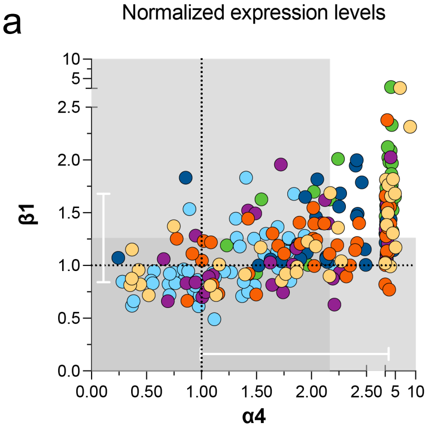

b

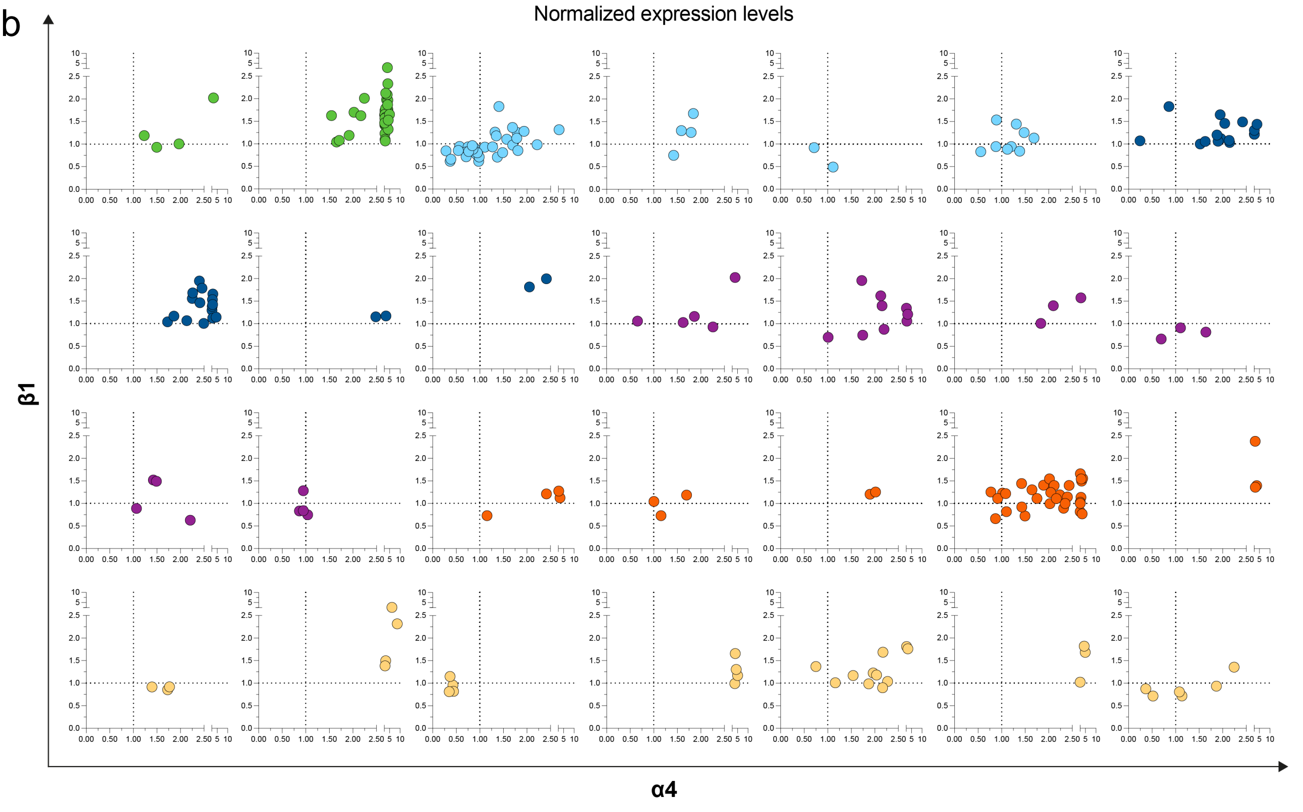

Supplementary Fig.3 α4β1 (VLA-4) phenotype of clonally expanded cells harboring inducible and translation-competent proviruses. Levels of expression of α4 and β1 are represented by the ratio between the fluorescence intensity of a cellular marker on each p24+ single sorted-cell and the mean fluorescence intensity of this marker on all CD4+ T cells from the same participant. A normalized expression level above or below 1 (dotted line) reflects a higher or lower expression of this marker on a given p24+ cell compared to all CD4+ T cells, respectively. Each dot represents a single-sorted cell and is color-coded by participant. **a** Normalized double-expression levels of α4β1 for all clonally expanded p24+ cells. Grey bars indicate the mean normalized expression level for each marker with its standard deviation in white. **b** Normalized double-expression levels of α4β1 of individual p24-expressing cells belonging to each individual clone.

Supplementary Fig. 4

a

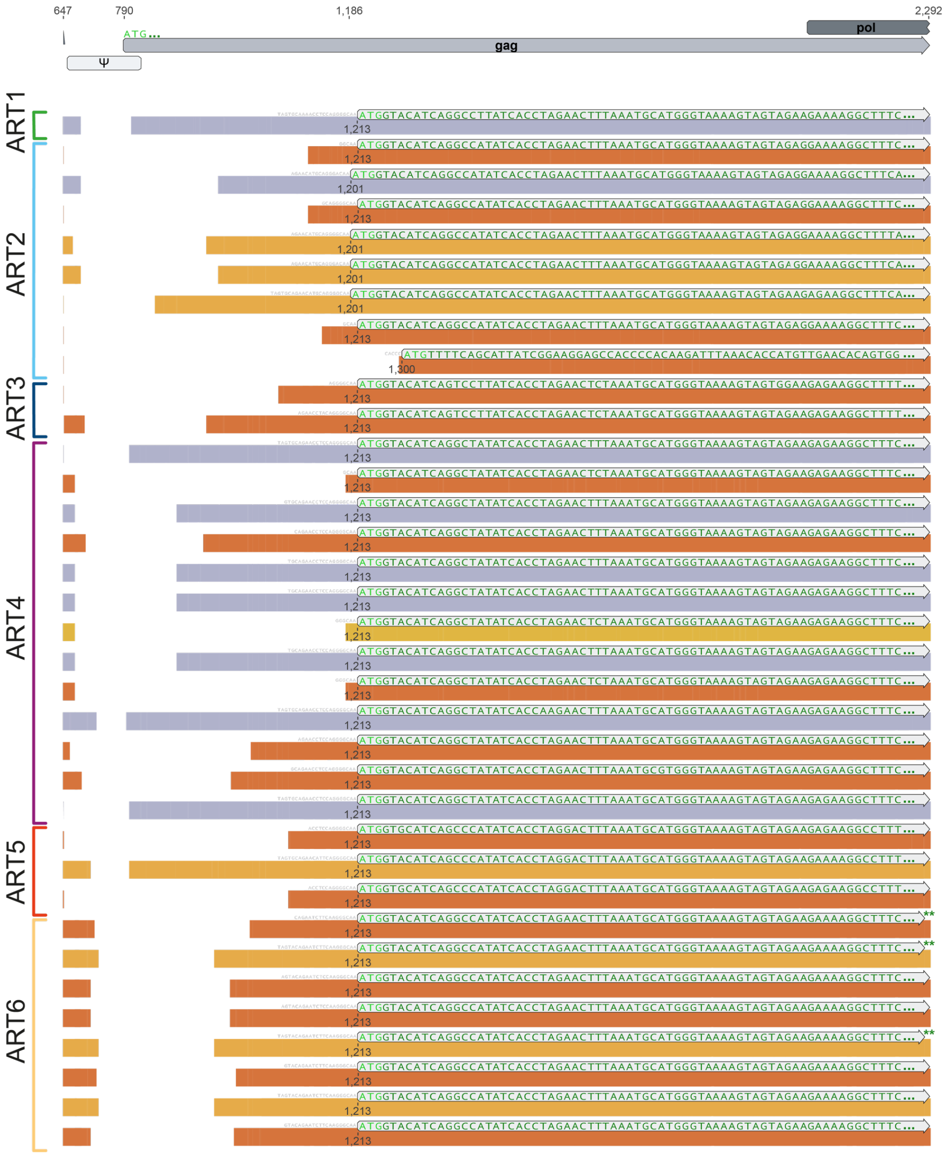

b

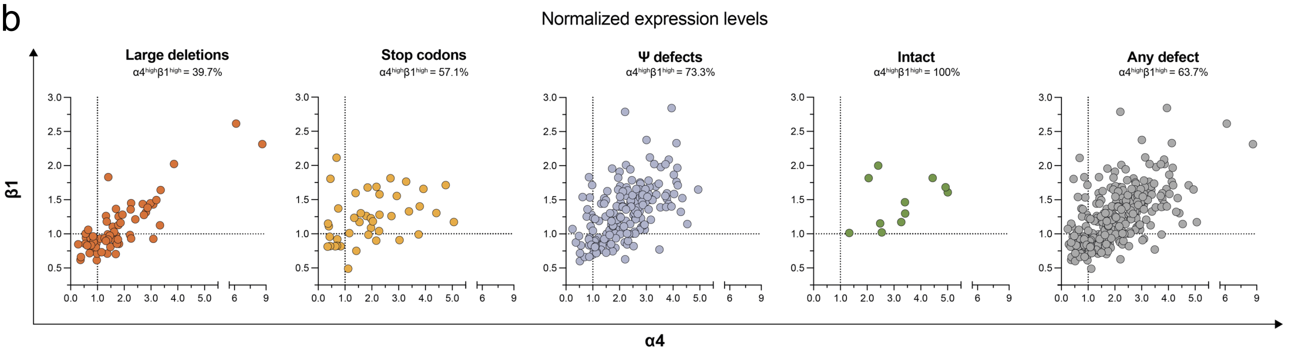

Supplementary Fig.4 Additional p24+ cells integrity analysis. a Alternative ATG start codon in the *gag* ORF of 35 unique HIV sequences retrieved in p24+ cells from 6 ART-suppressed participants. Initial start codon (HXB2 position 790), p24 region (HXB2 position 1,186) and "later *gag* start codon" (HXB2 equivalent position) are indicated. **b** Normalized double-expression levels of α4β1 of individual p24-expressing cells based on their proviral integrity. Frequencies of cells expressing high levels of both α4 and β1 in each integrity group are indicated at the top.

Supplementary Fig. 5

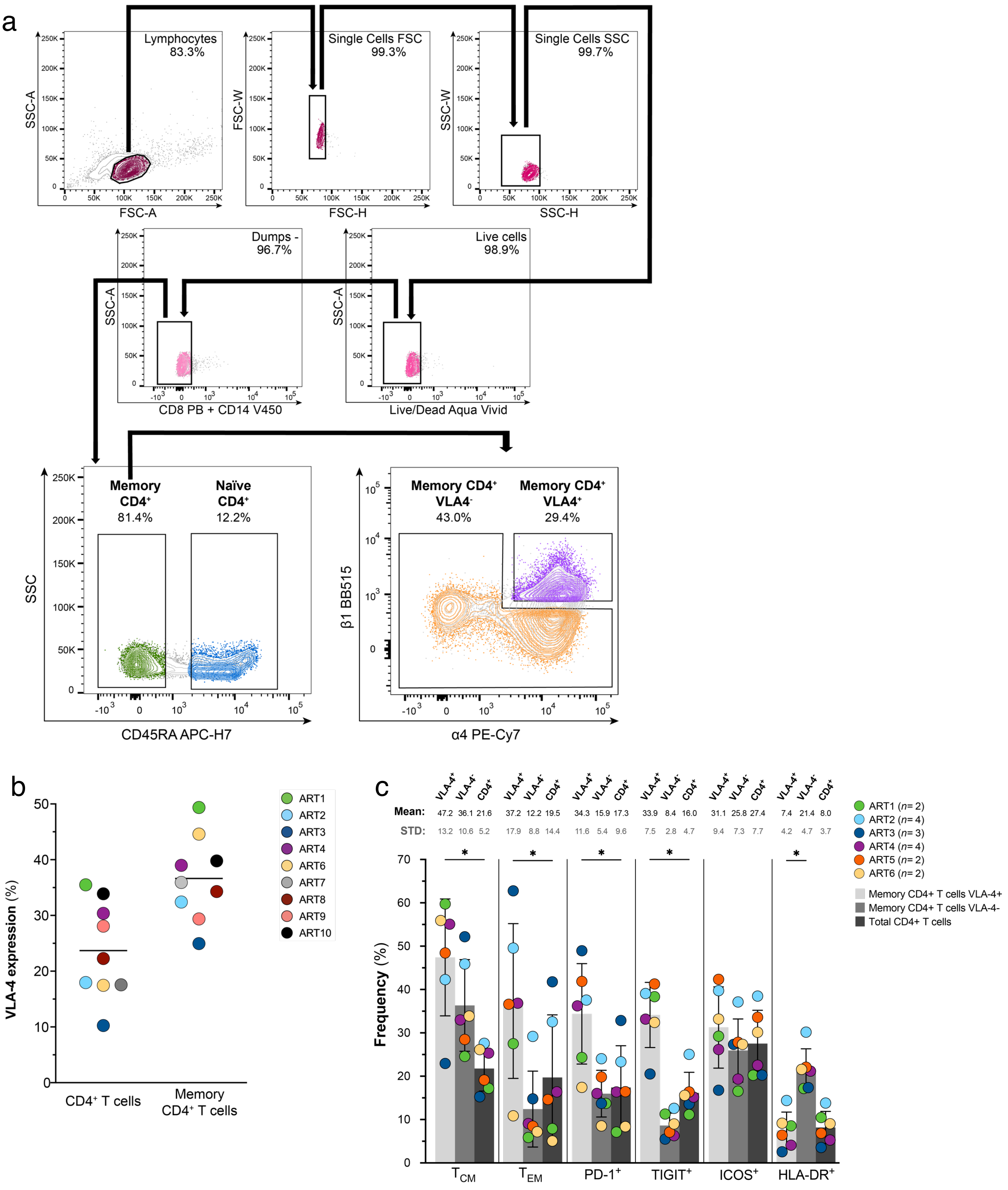

Supplementary Fig.5 α4β1 cell-sorting strategy. a Gating strategy for memory CD4+ T VLA-4+ cells sorting. Contour plots from a representative participant are shown. Memory CD4+ T cells expressing VLA-4 (VLA-4+: CD45RA-α4^{high} β1^{high}; purple contour plot) or not (VLA-4-: CD45RA-α4$^{low/-}$ β1$^{low/-}$; orange contour plot) were sorted by flow cytometry. **b** Frequencies of CD4+ T cells and memory CD4+ T cells expressing VLA-4 in the blood of 9 ART-suppressed participants. **c** Frequencies of memory CD4+ T cells expressing VLA-4 (α4+β1+; light grey bars), VLA-4- (α4- and/or β1-; grey bars) and total CD4+ T cells (dark grey bars) displaying a T_{CM} or T_{EM} phenotype, and expressing PD-1, TIGIT, ICOS and HLA-DR, in the blood of 6 ART-suppressed participants. Mean frequencies and standard deviations are indicated at the top of the graph. Mean and standard deviation (STD) for each population are indicated at the top. Differences in frequencies between cell populations were assessed by the non-parametric two-tailed Wilcoxon paired t-test (*: p = 0.0312).
