# Supplementary material for: Phenotypic characterization of single CD4+ T cells harboring genetically intact and inducible HIV genomes
Source: Nat Commun. 2023 Feb 27;14:1115. doi: 10.1038/s41467-023-36772-x (PMC9971253; doi:10.1038/s41467-023-36772-x)
Supplement: Supplementary file 3 — Reporting Summary [file 41467_2023_36772_MOESM3_ESM.pdf]

nature portfolio

Corresponding author(s): Nicolas Chomont

Last updated by author(s): Jan 17, 2023

Reporting Summary

Nature Portfolio wishes to improve the reproducibility of the work that we publish. This form provides structure for consistency and transparency in reporting. For further information on Nature Portfolio policies, see our Editorial Policies and the Editorial Policy Checklist.

Statistics

For all statistical analyses, confirm that the following items are present in the figure legend, table legend, main text, or Methods section.

n/a	Confirmed	
☐	☒	The exact sample size (*n*) for each experimental group/condition, given as a discrete number and unit of measurement
☐	☒	A statement on whether measurements were taken from distinct samples or whether the same sample was measured repeatedly
☐	☒	The statistical test(s) used AND whether they are one- or two-sided<br>*Only common tests should be described solely by name; describe more complex techniques in the Methods section.*
☒	☐	A description of all covariates tested
☒	☐	A description of any assumptions or corrections, such as tests of normality and adjustment for multiple comparisons
☐	☒	A full description of the statistical parameters including central tendency (e.g. means) or other basic estimates (e.g. regression coefficient) AND variation (e.g. standard deviation) or associated estimates of uncertainty (e.g. confidence intervals)
☐	☒	For null hypothesis testing, the test statistic (e.g. *F*, *t*, *r*) with confidence intervals, effect sizes, degrees of freedom and *P* value noted<br>*Give P values as exact values whenever suitable.*
☒	☐	For Bayesian analysis, information on the choice of priors and Markov chain Monte Carlo settings
☒	☐	For hierarchical and complex designs, identification of the appropriate level for tests and full reporting of outcomes
☐	☒	Estimates of effect sizes (e.g. Cohen's *d*, Pearson's *r*), indicating how they were calculated

Our web collection on statistics for biologists contains articles on many of the points above.

Software and code

Policy information about availability of computer code

Data collection	BD FACSDiva software v9.0.1, Image Lab v6.1.0
Data analysis	FlowJo v10.5.3, Prism v9.3.0 (GraphPad Software), Lima PacBio software v2.0.0, Geneious Prime v2021.1.1, FigTree v1.4.4, IQ-Tree2

For manuscripts utilizing custom algorithms or software that are central to the research but not yet described in published literature, software must be made available to editors and reviewers. We strongly encourage code deposition in a community repository (e.g. GitHub). See the Nature Portfolio guidelines for submitting code & software for further information.

Data

Policy information about availability of data

All manuscripts must include a data availability statement. This statement should provide the following information, where applicable:

- Accession codes, unique identifiers, or web links for publicly available datasets
- A description of any restrictions on data availability
- For clinical datasets or third party data, please ensure that the statement adheres to our policy

Sequences are available in GenBank, reference codes ON816029 to ON816663 (https://www.ncbi.nlm.nih.gov/popset?DbFrom=nuccore&Cmd=Link&LinkName=nuccore_popset&IdsFromResult=2306699925). Other datasets are available from the corresponding authors upon request.

Human research participants

Policy information about studies involving human research participants and Sex and Gender in Research.

Reporting on sex and gender	All participants were male living with HIV under suppressive ART.
Population characteristics	Participant's characteristics are summarized in Table 1. Briefly, all participants are men aged between 36 and 67 years old at the time of leukapheresis. They all have undetectable viral load (<40 copies per ml). Median time on ART is 12 years and CD4 T cell count is 731 cells per µl.
Recruitment	All participants were recruited at the McGill University Health Centre in Montréal. Dr. Jean-Pierre Routy recruited all participants in the study named "The Use of Leukapheresis to Support HIV Persistence Studies: HIV+ patients". According to inclusion and exclusion criteria of the study, candidates were offered to participate to this study and signed the Information and Consent Document. For feasibility of this sub-study, only participants with detectable HIV reservoirs by HIV-Flow were included.
Ethics oversight	All participants were adults and signed informed consent forms approved by the McGill University Health Centre and the Centre Hospitalier de l'Université de Montréal review boards (MP-CUSM-15-303).

Note that full information on the approval of the study protocol must also be provided in the manuscript.

Field-specific reporting

Please select the one below that is the best fit for your research. If you are not sure, read the appropriate sections before making your selection.

☒ Life sciences ☐ Behavioural & social sciences ☐ Ecological, evolutionary & environmental sciences

For a reference copy of the document with all sections, see nature.com/documents/nr-reporting-summary-flat.pdf

Life sciences study design

All studies must disclose on these points even when the disclosure is negative.

Sample size	Ten HIV-infected ART-treated participants were recruited in the study. Sample size was not calculated based on power analysis and was determined by sample availability.
Data exclusions	qVOA cultures that got contaminated during cell culture were excluded from the analysis, and cell-sorting of these samples were repeated. We also excluded one proviral sequence obtained from a p24+ cell and which had a deletion of the entire gag gene, suggesting an issue during the sequencing process.
Replication	All data acquired are reported, therefore the reproducibility can be estimated within the manuscript. Every sample has been sorted multiple times in independant sorting experiment.
Randomization	No randomization was performed. Randomization is not applicable to the study because the study has no control group.
Blinding	Blinding was not performed. Blinding is not applicable to the study.

Reporting for specific materials, systems and methods

We require information from authors about some types of materials, experimental systems and methods used in many studies. Here, indicate whether each material, system or method listed is relevant to your study. If you are not sure if a list item applies to your research, read the appropriate section before selecting a response.

Materials & experimental systems

n/a	Involved in the study	
☐	☒	Antibodies
☐	☒	Eukaryotic cell lines
☒	☐	Palaeontology and archaeology
☒	☐	Animals and other organisms
☒	☐	Clinical data
☒	☐	Dual use research of concern

Methods

n/a	Involved in the study	
☒	☐	ChIP-seq
☐	☒	Flow cytometry
☒	☐	MRI-based neuroimaging

Antibodies

Antibodies used	Live/Dead Aqua Cell Stain (ThermoFisher Scientific cat.L34957; 0.0025 µl per 100 µl),<br>CD45RA APC-H7 (clone HI100; BD cat.560674; 2 µl per 100 µl),<br>CCR7 BB700 (clone 3D12; BD cat.566437; 1 µl per 100 µl),<br>PD-1 BV605 (clone EH12.2H7; Biolegend cat.329924; 5 µl per 100 µl),<br>TIGIT eF450 (clone MBSA43; eBioscience cat.48-9500-42; 2 µl per 100 µl),<br>HLA-DR AlexaFluor700 (clone G46-6; BD cat. 560743; 0.5 µl per 100 µl),<br>ICOS BV785 (clone C398.4A; Biolegend cat.313534; 4 µl per 100 µl),<br>CD8 PB (clone RPA-T8; BD cat.558207; 2 µl per 100 µl),<br>CD14 V450 (clone MϕP9; BD cat.560349; 1 µl per 100 µl),<br>α4/CD49d PE-Cy7 (clone 9F10; Biolegend cat.304313; 0.5 µl per 100 µl)<br>β1/CD29 BB515 (clone MAR4; BD cat.564565; 4 µl per 100 µl),<br>p24 APC (clone 28B7; MediMabs cat.MM-0289-APC; 0.1 µl per 100 µl),<br>p24-PE (clone KC57; Beckman Coulter cat.6604667; 0.1 µl per 100 µl),<br>CD3 (clone OKT3; Biolegend cat. 317302; 2.5 µg/ml),<br>CD28 (clone CD28.2; Biolegend cat. 302902; 1 µg/ml)
Validation	All antibodies are commercially available. Live/Dead Aqua Cell Stain, CD45RA APC-H7, CCR7 B700, PD-1 BV605, TIGIT eF450, HLA-DR AF700, ICOS BV785, CD8 PB, CD14 V450, α4 PE-Cy7, β1 BB515, p24 APC, p24 PE are all tested by manufacturers for flow cytometry application in human samples. CD3 OKT3 is tested by manufacturer for T cell activation (human) and CD28 CD28.2 is indicated as "reported in litterature" for costimulation by manufacturer (human).<br>We performed titrations to determine optimal antibody concentrations for each antibody used in this study.

Eukaryotic cell lines

Policy information about cell lines and Sex and Gender in Research

Cell line source(s)	ACH-2 cell line (The following reagent was obtained through the NIH HIV Reagent Program, Division of AIDS, NIAID, NIH: ACH-2 Cells, ARP-349, contributed by Dr. Thomas Folks: https://www.hivreagentprogram.org/Catalog/HRPCellLines/ARP-349.aspx)<br>MOLT-4 CCR5+ cell line (The following reagent was obtained through the NIH HIV Reagent Program, Division of AIDS, NIAID, NIH: MOLT-4 CCR5+ Cells, ARP-4984, contributed by Dr. Masanori Baba, Dr. Hiroshi Miyake, and Dr. Yuji Iizaw. https://www.hivreagentprogram.org/Catalog/HRPCellLines/ARP-4984.aspx)
Authentication	ARP-349 is a T-cell clone of A3.01 cells that are CD4-, CD5+, transferrin receptor+ and Leu-1+, with one integrated proviral copy of latent human immunodeficiency virus type 1 (HIV-1) LAV.<br>ARP-4984 contain a CCR5 expression plasmid was transfected into MOLT-4 cells and stable transfectants were selected in culture medium containing G418 (1 mg per mL).<br>No technical informations about the authentification method used by the HIV Reagent Program is provided in their website.
Mycoplasma contamination	HIV reagent program states that the cell line has tested negative for bacteria, fungi and mycoplasma .
Commonly misidentified lines (See ICLAC register)	None

Flow Cytometry

Plots

Confirm that:

- [x] The axis labels state the marker and fluorochrome used (e.g. CD4-FITC).
- [x] The axis scales are clearly visible. Include numbers along axes only for bottom left plot of group (a 'group' is an analysis of identical markers).
- [x] All plots are contour plots with outliers or pseudocolor plots.
- [x] A numerical value for number of cells or percentage (with statistics) is provided.

Methodology

Sample preparation	PBMCs were isolated from leukapheresis product by Ficoll density gradient centrifugation. CD4+ T cells were enriched by negative magnetic selection using the EasySep Human CD4 T Cell Enrichment Kit (StemCell Technology, 19052).
Instrument	Flow cytometry cell sortings were collected using a FACSAria II cell sorter (BD Biosciences).
Software	Acquisition was performed with BD FACSDiva software and analysis was performed with FlowJo v10.
Cell population abundance	p24+ cells frequencies for each participant are indicated in Table 1.

Gating strategy	Fig.1b shows the gating strategy for index cell-sorting of p24+ and p24- populations, with an additional detailed gating strategy in Suppl. 1b. Suppl.3a shows gating strategy for VLA4+ memory CD4+ T cells.

[x] Tick this box to confirm that a figure exemplifying the gating strategy is provided in the Supplementary Information.
