# Supplementary material for: Phenotypic characterization of single CD4+ T cells harboring genetically intact and inducible HIV genomes
Source: Nat Commun. 2023 Feb 27;14:1115. doi: 10.1038/s41467-023-36772-x (PMC9971253; doi:10.1038/s41467-023-36772-x)
Supplement: Supplementary file 4 — Source Data [file 41467_2023_36772_MOESM4_ESM.pdf]

Figure 3a

	p24+	p24-
	# of proviral sequence	
Unique	65	163
Clonal	243	163

Figure 3b

Copies per clone	p24+							p24-						
	# of proviral sequence							# of proviral sequence						
	Total	ART1	ART2	ART3	ART4	ART5	ART6	Total	ART1	ART2	ART3	ART4	ART5	ART6
2 copies	3		1	1		1		27	9		4	5	3	6
3 copies	7			1	2	2	2	8	1		1	5	1	
4 copies	6	1	1		1		3	4	2			1		1
5 copies	2				2			2			1		1	
6 copies	1						1	1	1					
9 copies	2		1		1			0						
12 copies	1						1	0						
15 copies	0							1						1
16 copies	1			1				0						
17 copies	1			1				0						
32 copies	1					1		0						
33 copies	1		1					0						
37 copies	0							1		1				
44 copies	1	1						0						

Figure 3c

	ART1 # of proviral sequence		ART2 # of proviral sequence		ART3 # of proviral sequence		ART4 # of proviral sequence		ART5 # of proviral sequence		ART6 # of proviral sequence	
Clone ID	p24+	p24-	p24+	p24-	p24+	p24-	p24+	p24-	p24+	p24-	p24+	p24-
I		2		37		2		2		2		2
II		2	33			2		3		2		2
III		4	4			3		4		2		4
IV		2	2			5		2	4	1		2
V		2	9			2		3		1		2
VI		2	1			2		3	3			15
VII		2			17		1	2				2
VIII		6			16	1	5	3	2		3	
IX		4			3		9	1	32	3	4	
X		2			2		3		1	5	4	1
XI		2					3	2	3		1	1
XII		3					5	3				2
XIII		2					4	2			4	1
XIV	4	1					1	1			12	
XV	44	1									3	
XVI											6	
Unique	4	31	6	17	12	36	10	20	7	8	22	42

Figure 4a

PID	PD-1 (FI/StimPop_MFI)	TIGIT (FI/StimPop_MFI)	HLA-DR (FI/StimPop_MFI)	ICOS (FI/StimPop_MFI)	β1 (FI/StimPop_MFI)	α4 (FI/StimPop_MFI)	CD45RA (FI/StimPop_MFI)	CCR7 (FI/StimPop_MFI)
ART1	0,953	0,646	1,28	1,001	1,231	1,19	1,261	0,329
ART1	1,061	1,029	0,706	0,909	1,497	0,929	1,286	0,498
ART1	0,998	1,013	1,133	0,826	1,969	1,006	0,465	0,509
ART1	1,018	1,029	1,024	0,715	3,295	2,024	0,33	0,304
ART1	1,974	1,375	1,523	1,39	4,125	2,331	0,331	0,599
ART1	1,343	1,227	1,007	0,826	2,742	1,415	0,574	0,708
ART1	1,428	1,257	1,124	0,963	2,762	1,444	0,554	0,667
ART1	1,437	1,376	1,189	0,965	3,772	1,595	0,557	0,352
ART1	1,365	1,427	1,524	0,83	3,376	1,575	0,459	0,677
ART1	1,408	1,42	0,723	1,107	4,125	1,763	0,469	0,629
ART1	1,706	1,539	1,091	1,191	2,822	1,687	0,549	0,678
ART1	1,672	1,506	0,69	0,926	3,05	1,661	0,47	0,534
ART1	1,572	1,4	1,148	0,753	3,351	1,696	0,712	0,336
ART1	1,245	1,148	1,369	1,008	2,645	1,64	0,284	0,624
ART1	1,081	1,293	1,384	0,776	3,541	1,979	0,541	0,584
ART1	1,195	1,189	1,127	0,914	3,157	1,499	0,608	0,52
ART1	0,942	0,909	0,808	1,011	2,022	1,699	0,525	0,761
ART1	1,041	1,036	1,159	1,127	2,167	1,624	0,419	0,511
ART1	0,919	1,449	0,873	0,927	4,161	1,968	0,406	0,571
ART1	1,179	0,804	1,309	1,296	3,466	1,737	0,499	0,469
ART1	1,019	1,261	0,493	1,403	3,116	1,689	0,88	0,556
ART1	0,938	0,822	1,044	1,076	1,918	1,187	0,593	0,469
ART1	1,043	1,242	1,014	1,001	2,241	2,01	0,443	0,489
ART1	0,983	0,953	0,996	0,81	3,528	1,39	0,433	0,384
ART1	0,98	1,224	0,815	0,88	3,841	2,089	0,554	0,488
ART1	0,984	1,262	0,571	0,752	2,802	1,229	0,332	0,444
ART1	1,069	1,665	0,73	0,897	4,174	1,713	0,305	0,64
ART1	1,035	1,165	0,968	0,729	4,036	1,585	0,427	0,589
ART1	1,044	1,361	1,1	0,899	3,945	2,845	0,381	0,38
ART1	1,071	1,247	1,055	0,83	2,885	1,514	0,227	0,326
ART1	0,886	0,877	0,8	1,018	1,545	1,627	0,216	0,621
ART1	0,946	1,232	0,777	0,629	3,769	1,514	0,575	0,665
ART1	1,139	1,314	0,805	1,027	2,977	1,467	0,241	0,329
ART1	0,952	1,374	1,227	0,795	2,78	1,737	0,411	0,185
ART1	1,009	1,004	0,975	1,091	3,084	1,783	0,231	0,481
ART1	1,066	1,469	0,911	0,742	3,134	2,122	0,326	0,401
ART1	1,036	1,284	0,875	0,827	2,748	1,651	0,46	0,417
ART1	1,055	1,624	1,006	1,118	4,028	1,866	0,238	0,389
ART1	1,094	1,08	1,169	0,849	3,084	1,583	0,216	0,485
ART1	1,141	0,979	0,627	0,723	2,722	1,566	0,575	0,282
ART1	1,003	1,096	1,249	0,889	1,651	1,04	0,383	0,346
ART1	1,01	1,152	0,912	0,803	4,29	1,323	0,475	0,394
ART1	1,037	1,089	0,967	0,868	2,813	1,098	0,364	0,564
ART1	1,062	1,328	1,054	1,419	4,938	1,651	0,281	0,44
ART1	1,043	1,198	0,78	0,947	1,709	1,075	0,309	0,395
ART1	1,037	1,156	0,917	1,1	2,614	1,465	0,423	0,271
ART1	1,002	1,244	0,696	0,703	2,942	1,064	0,525	0,736
ART1	1,184	1,333	0,545	1,195	4,345	1,525	0,318	0,494
ART2	1,113	1,215	1,785	0,926	0,368	0,618	0,607	0,659
ART2	1,146	1,249	1,395	0,764	0,964	0,862	0,753	0,909
ART2	1,171	0,96	1,152	0,817	1,321	1,262	0,361	0,348
ART2	1,327	0,938	1,258	0,879	1,572	1,105	0,312	0,373
ART2	1,166	1,14	0,23	0,988	1,697	0,976	0,949	1,203
ART2	0,969	1,172	1,221	0,869	0,568	0,945	0,673	1,085
ART2	0,984	0,898	1,198	0,774	1,405	1,831	1,186	0,447
ART2	1,004	1,063	1,328	1,149	0,927	0,77	0,267	2,284
ART2	0,993	0,795	0,97	1,142	1,348	1,179	0,598	1,649
ART2	1,007	0,849	0,559	0,79	0,974	0,614	0,815	1,49
ART2	0,942	0,99	0,885	0,817	0,749	0,934	0,564	1,649
ART2	0,789	0,966	0,993	0,999	0,985	0,712	0,555	2,193
ART2	0,784	0,78	0,507	1,049	1,792	1,254	0,489	0,938
ART2	1,038	0,952	0,815	1,101	0,701	0,823	0,815	0,573
ART2	0,916	0,95	1,098	0,834	1,104	0,93	0,714	1,925
ART2	1	0,982	1,268	0,869	0,872	0,926	0,491	0,494
ART2	1,088	0,8	1,26	0,972	2,216	0,984	0,977	0,641
ART2	0,908	0,882	0,443	0,937	1,692	1,365	0,663	1,281
ART2	0,927	0,877	1,06	0,766	0,709	0,717	0,781	0,881
ART2	1,017	0,89	1,007	1,06	1,37	0,71	0,603	1,181
ART2	1,031	1,044	0,893	0,836	0,542	0,854	1,022	1,09
ART2	0,944	0,871	1,424	1,238	1,484	0,808	0,272	0,682
ART2	1	1,483	0,971	0,913	0,281	0,847	2,031	1,219
ART2	0,955	1,272	0,816	1,109	1,269	0,936	0,746	1,252
ART2	0,902	0,727	0,954	0,92	1,782	1,128	0,608	1,312
ART2	0,889	0,778	1,058	0,82	0,944	0,806	0,728	0,592
ART2	1,012	1,112	1,388	0,796	0,853	0,882	0,994	0,998
ART2	0,977	0,773	0,921	1,226	2,812	1,317	0,741	1,166
ART2	0,976	0,878	1,277	0,568	1,938	1,281	0,774	1,083
ART2	0,996	0,93	1,181	1,123	0,763	0,833	0,783	0,455

ART2	0,914	0,773	0,694	0,775	0,383	0,661	0,649	1,247
ART2	1,089	1,252	1,509	1,319	0,838	0,963	0,81	0,709
ART2	1,025	1,192	0,946	0,716	1,808	0,854	0,803	0,721
ART2	1,027	0,984	1,008	0,804	1,793	1,26	0,752	0,895
ART2	0,869	0,678	0,908	0,808	1,586	1,3	0,834	0,746
ART2	1,048	0,892	0,991	0,634	1,842	1,677	0,723	1,588
ART2	0,805	0,636	1,201	0,798	1,423	0,751	0,72	0,937
ART2	1,278	0,966	1,517	0,85	0,712	0,921	0,282	0,884
ART2	1,017	0,719	0,681	0,663	1,117	0,49	1,071	1,438
ART2	1,099	1,042	1,188	1,004	1,206	0,944	0,989	0,528
ART2	0,951	1,012	1,131	0,907	0,891	1,534	0,988	0,448
ART2	1,009	1,213	0,827	0,956	1,312	1,444	0,656	0,514
ART2	0,954	0,859	1,029	0,814	1,122	0,885	0,784	0,937
ART2	0,918	0,892	1,181	0,914	0,88	0,945	0,707	0,43
ART2	0,932	0,979	1,287	0,775	1,689	1,133	0,829	1,046
ART2	0,984	0,786	1,269	1,084	1,478	1,254	0,525	1,286
ART2	1,004	0,847	1,017	0,863	0,557	0,831	0,612	1,052
ART2	1	1,123	1,019	0,826	1,385	0,844	0,746	1,065
ART3	1,023	0,962	0,929	0,888	1,959	1,12	0,782	0,738
ART3	1,013	0,623	1,059	1,255	2,42	1,489	0,699	0,358
ART3	0,995	1,005	1,098	0,816	0,857	1,832	0,645	1,785
ART3	0,895	1,085	1,255	1,609	0,243	1,072	0,79	1,567
ART3	0,935	0,952	1,138	0,707	1,944	1,649	0,635	0,704
ART3	0,973	0,846	0,984	0,86	1,521	1,001	0,576	0,87
ART3	0,912	1,025	0,757	1,134	2,041	1,458	0,557	0,625
ART3	0,952	0,983	0,789	0,782	1,628	1,056	0,537	0,909
ART3	0,919	0,916	0,799	0,827	2,145	1,033	0,81	0,781
ART3	1,017	0,968	1,277	0,899	2,59	1,216	0,714	0,292
ART3	1,004	1,034	0,904	0,753	1,899	1,061	0,674	0,464
ART3	0,92	0,909	0,974	0,849	2,601	1,217	0,57	0,595
ART3	0,949	0,999	0,887	0,867	2,558	1,303	0,623	0,392
ART3	0,959	0,805	0,843	0,569	3,834	1,437	0,956	0,566
ART3	1,053	1,018	0,994	0,901	2,136	1,081	0,941	0,341
ART3	1,011	0,8	1,184	0,992	1,877	1,2	0,701	0,459
ART3	0,893	0,907	1,016	0,731	2,495	1,005	0,683	0,233
ART3	1,075	0,808	1,443	0,942	2,403	1,947	0,698	0,309
ART3	0,899	0,956	1,142	0,858	2,252	1,558	0,811	0,819
ART3	0,875	0,763	0,942	0,96	2,968	1,656	0,785	0,588
ART3	1,054	0,986	1,16	0,985	2,768	1,533	0,66	0,634
ART3	1,042	0,91	0,804	0,815	2,589	1,296	1,136	0,692
ART3	0,919	0,827	0,796	1,024	1,727	1,04	0,739	0,792
ART3	0,921	0,753	0,696	0,754	2,261	1,683	0,628	0,804
ART3	0,961	0,948	1,356	0,74	2,893	1,124	0,607	0,375
ART3	0,924	1,053	1,678	1,105	2,133	1,066	0,285	0,425
ART3	1,125	1,043	0,905	0,917	2,413	1,465	1,305	0,616
ART3	1,025	0,91	1,245	0,975	2,699	1,385	0,388	0,622
ART3	0,991	1,054	1,655	1,138	2,458	1,787	0,355	0,519
ART3	0,923	0,887	1,114	0,832	1,861	1,167	0,95	0,616
ART3	0,983	1,03	1,237	0,993	2,875	1,424	0,899	0,693
ART3	0,901	1,069	1,152	0,873	4,534	1,144	0,577	0,719
ART3	0,95	1,254	1,177	0,86	2,486	1,153	0,816	0,313
ART3	1,01	0,731	1,749	0,741	3,273	1,172	0,5	0,558
ART3	1,05	1,03	1,247	1,108	2,413	1,998	0,577	0,859
ART3	0,959	1,039	1,052	0,907	2,053	1,816	0,675	0,545
ART4	0,924	1,106	0,829	0,858	0,657	1,06	0,486	0,737
ART4	1,095	1,218	1,232	1,052	1,625	1,028	0,378	0,64
ART4	1,146	0,934	0,639	0,751	2,257	0,931	0,453	1,146
ART4	1,096	1,34	0,843	0,691	3,861	2,025	0,89	0,595
ART4	1,045	1,272	0,937	0,869	1,863	1,162	0,543	0,393
ART4	1,336	1,123	1,248	0,878	2,151	1,399	0,239	0,433
ART4	1,043	0,985	1,035	0,813	2,756	1,058	0,269	0,773
ART4	1,619	1,158	1,135	0,837	2,194	0,876	0,695	0,445
ART4	1,326	1,05	1,242	0,831	2,594	1,344	0,727	0,243
ART4	1,041	1,039	0,789	0,802	1,007	0,699	0,657	0,507
ART4	1,147	1,166	1,595	0,842	1,722	1,957	0,749	0,718
ART4	1,02	0,854	1,221	1,044	1,741	0,746	0,423	0,589
ART4	0,979	0,841	0,959	0,957	2,124	1,62	0,514	0,642
ART4	0,985	1,163	1,138	0,821	3,169	1,209	0,492	0,206
ART4	1,025	0,959	1,031	0,849	1,832	1,007	0,392	0,491
ART4	1,158	0,963	1,146	0,973	2,097	1,401	0,744	0,338
ART4	1,024	0,914	0,919	0,815	2,864	1,575	0,587	0,498
ART4	1,106	1,158	1,044	0,97	1,643	0,816	0,864	0,598
ART4	0,966	0,893	1,521	0,806	1,104	0,909	0,684	0,537
ART4	1,157	1,044	1,466	0,803	0,694	0,661	0,466	0,31
ART4	0,982	1,035	1,583	1,007	1,424	1,521	0,274	0,529
ART4	0,954	0,93	1,359	0,86	1,49	1,493	0,288	0,825
ART4	1,031	1,254	2,078	0,857	2,209	0,628	0,472	0,415
ART4	0,957	1,098	1,029	0,84	1,066	0,891	0,516	0,665
ART4	1,165	1,201	0,98	0,879	0,863	0,833	0,437	0,595
ART4	1,256	1,145	1,23	0,989	1,039	0,749	0,493	0,687
ART4	1,052	1,491	0,821	0,984	0,946	1,282	0,643	0,555
ART4	1,016	1,238	1,127	0,752	0,946	0,84	0,435	0,62

ART5	0,968	1,234	1,047	0,732	2,416	1,213	0,333	0,448
ART5	0,965	1,157	0,826	0,903	3,337	1,126	0,505	0,619
ART5	1,068	1,372	1,4	0,74	2,734	1,277	0,448	0,399
ART5	1,048	0,609	1,496	1,127	1,154	0,731	0,36	0,395
ART5	0,991	0,829	0,767	0,971	1,004	1,046	0,458	0,957
ART5	1,048	0,609	1,496	1,127	1,154	0,731	0,36	0,395
ART5	1,085	1,07	0,94	1,132	1,698	1,186	0,359	0,656
ART5	1,286	1,244	1,143	0,689	1,899	1,206	0,225	1,221
ART5	1,491	1,222	0,942	0,727	2,018	1,255	0,491	0,835
ART5	1,01	1,029	1,286	0,641	1,497	0,724	0,519	0,56
ART5	0,951	1,111	0,847	0,748	1,646	1,303	0,361	0,674
ART5	1,243	1,04	0,95	0,773	3,229	1,498	0,518	0,699
ART5	0,975	1,686	0,666	0,525	1,751	1,111	0,632	0,559
ART5	0,979	1,592	0,856	0,797	0,774	1,254	0,683	0,349
ART5	1,095	1,08	1,612	0,829	2,025	1,395	0,236	0,488
ART5	1,055	1,304	1,194	0,662	1,891	1,406	0,497	0,558
ART5	1,061	1,131	1,133	0,686	1,103	0,819	0,281	0,443
ART5	0,953	1,261	1,187	0,743	2,933	1,136	0,454	0,656
ART5	0,967	1,225	0,877	0,635	1,02	1,232	0,593	0,569
ART5	1,009	1,76	0,948	0,993	2,236	1,191	0,755	0,69
ART5	1	1,906	1,117	0,825	2,557	1,03	0,412	0,49
ART5	1,084	0,991	1,423	0,692	2,526	0,82	0,669	0,259
ART5	1,052	1,389	1,275	0,682	2,583	1,004	0,47	0,428
ART5	1,07	1,461	0,93	0,65	2,025	0,995	0,492	0,58
ART5	1,13	1,041	1,272	0,869	2,017	1,546	0,484	0,398
ART5	1,104	1,324	1,327	0,663	2,117	1,397	0,584	0,532
ART5	0,949	1,408	1,112	0,801	3,711	1,556	0,426	0,823
ART5	1,025	0,977	1,102	1,035	0,917	1,111	0,406	0,679
ART5	1,003	1,368	1,131	0,861	2,625	1,659	0,395	0,443
ART5	1,037	1,401	1,02	0,679	2,397	1,145	0,547	0,594
ART5	0,954	1,158	0,997	0,754	2,317	0,896	0,429	0,527
ART5	1,285	1,496	1,804	0,725	3,045	1,544	0,583	0,458
ART5	1,087	1,051	1,258	0,678	0,871	0,664	0,496	0,433
ART5	1,026	1,01	1,936	0,741	3,491	0,771	0,448	0,454
ART5	1,269	1,043	0,764	0,55	2,433	1,4	0,373	0,665
ART5	1,04	1,352	0,847	0,855	1,086	1,22	0,532	0,579
ART5	1,168	0,923	0,947	0,712	1,424	1,444	0,479	0,535
ART5	1,361	1,064	1,15	0,632	1,429	0,923	0,417	0,328
ART5	1,745	1,181	1,199	0,996	2,351	1	0,345	0,378
ART5	1,023	1,439	0,73	0,643	2,044	1,242	0,328	0,582
ART5	0,982	1,516	1,119	0,616	2,164	1,109	0,647	0,519
ART5	1,052	1,258	1,158	0,845	3,743	1,395	0,568	0,941
ART5	1,045	0,707	0,992	0,926	3,008	2,378	0,5	0,546
ART5	0,905	1,095	1,237	0,666	3,084	1,36	0,428	0,548
ART6	1,123	1,101	1,537	0,896	1,729	0,854	0,569	0,631
ART6	0,99	0,988	0,855	0,824	1,766	0,919	0,611	0,947
ART6	0,964	1,027	0,843	0,795	1,396	0,915	0,419	1,117
ART6	0,95	0,95	1,138	0,787	8,646	2,315	0,839	0,468
ART6	0,992	0,99	0,877	1,096	3,201	1,496	0,262	1,589
ART6	1,005	0,801	1,398	0,76	6,152	2,616	0,444	0,808
ART6	1,069	0,88	1,089	0,984	2,873	1,384	0,4	0,883
ART6	1,024	1,339	0,852	1,22	0,428	0,957	0,536	1,935
ART6	1,02	1,463	1,675	0,98	0,443	0,82	0,451	0,588
ART6	0,944	1,736	0,871	2,729	0,351	0,813	0,748	0,877
ART6	1,05	0,925	1,106	1,56	0,368	1,15	0,44	0,739
ART6	1,05	1,615	0,711	1,44	3,745	0,991	2,338	2,285
ART6	0,993	1,134	1,436	0,908	5,06	1,171	0,495	0,449
ART6	1,041	1,014	0,876	0,774	4,407	1,304	0,345	1,541
ART6	0,973	0,913	0,969	0,883	3,926	1,657	0,427	1,019
ART6	1	1,47	1,109	0,767	2,271	1,039	0,458	0,618
ART6	0,986	1,281	1,035	0,859	1,965	1,227	0,494	0,424
ART6	0,972	1,282	1,148	0,638	2,695	1,813	0,318	1,039
ART6	1,007	1,11	0,888	0,967	2,172	1,687	0,429	1,126
ART6	0,959	1,195	1,068	0,834	1,539	1,171	0,418	1,075
ART6	0,93	0,965	1,183	0,83	2,165	0,9	0,268	1,119
ART6	1,01	1,008	1,029	0,67	2,038	1,182	0,374	1,224
ART6	0,996	1,119	0,835	0,851	1,161	1,009	0,46	0,819
ART6	1,025	0,892	1,083	0,753	0,749	1,371	0,35	1,231
ART6	0,972	0,838	0,834	0,727	3,278	1,763	0,461	1,091
ART6	0,959	1,147	1,065	0,778	1,87	0,986	0,472	1,29
ART6	0,978	0,864	0,933	0,91	4,927	1,682	0,374	1,346
ART6	0,935	0,713	1,655	0,555	2,544	1,021	0,25	0,99
ART6	1,047	1,017	2,372	0,74	4,444	1,818	0,146	1,125
ART6	0,991	1,248	0,952	0,812	0,52	0,717	0,742	0,981
ART6	0,99	1,607	1,387	0,879	1,865	0,936	0,476	0,647
ART6	1,028	1,292	1,087	0,823	1,133	0,719	0,431	0,495
ART6	1,054	1,13	0,925	0,811	1,08	0,808	0,274	1,201
ART6	0,995	1,248	0,911	1,279	2,242	1,357	0,568	0,714
ART6	0,963	1,129	0,803	0,903	0,367	0,875	0,475	1,225

Figure 4b

PID	Clone ID	PD-1 (FI/StimPop_MFI)	TIGIT (FI/StimPop_MFI)	HLA-DR (FI/StimPop_MFI)	ICOS (FI/StimPop_MFI)	β1 (FI/StimPop_MFI)	α4 (FI/StimPop_MFI)	CD45RA (FI/StimPop_MFI)	CCR7 (FI/StimPop_MFI)
ART1	I	0,953	0,646	1,280	1,001	1,231	1,190	1,261	0,329
ART1	I	1,061	1,029	0,706	0,909	1,497	0,929	1,286	0,498
ART1	I	0,998	1,013	1,133	0,826	1,969	1,006	0,465	0,509
ART1	I	1,018	1,029	1,024	0,715	3,295	2,024	0,330	0,304
ART1	II	1,974	1,375	1,523	1,390	4,125	2,331	0,331	0,599
ART1	II	1,343	1,227	1,007	0,826	2,742	1,415	0,574	0,708
ART1	II	1,428	1,257	1,124	0,963	2,762	1,444	0,554	0,667
ART1	II	1,437	1,376	1,189	0,965	3,772	1,595	0,557	0,352
ART1	II	1,365	1,427	1,524	0,830	3,376	1,575	0,459	0,677
ART1	II	1,408	1,420	0,723	1,107	4,125	1,763	0,469	0,629
ART1	II	1,706	1,539	1,091	1,191	2,822	1,687	0,549	0,678
ART1	II	1,672	1,506	0,690	0,926	3,050	1,661	0,470	0,534
ART1	II	1,572	1,400	1,148	0,753	3,351	1,696	0,712	0,336
ART1	II	1,245	1,148	1,369	1,008	2,645	1,640	0,284	0,624
ART1	II	1,081	1,293	1,384	0,776	3,541	1,979	0,541	0,584
ART1	II	1,195	1,189	1,127	0,914	3,157	1,499	0,608	0,520
ART1	II	0,942	0,909	0,808	1,011	2,022	1,699	0,525	0,761
ART1	II	1,041	1,036	1,159	1,127	2,167	1,624	0,419	0,511
ART1	II	0,919	1,449	0,873	0,927	4,161	1,968	0,406	0,571
ART1	II	1,179	0,804	1,309	1,296	3,466	1,737	0,499	0,469
ART1	II	1,019	1,261	0,493	1,403	3,116	1,689	0,880	0,556
ART1	II	0,938	0,822	1,044	1,076	1,918	1,187	0,593	0,469
ART1	II	1,043	1,242	1,014	1,001	2,241	2,010	0,443	0,489
ART1	II	0,983	0,953	0,996	0,810	3,528	1,390	0,433	0,384
ART1	II	0,980	1,224	0,815	0,880	3,841	2,089	0,554	0,488
ART1	II	0,984	1,262	0,571	0,752	2,802	1,229	0,332	0,444
ART1	II	1,069	1,665	0,730	0,897	4,174	1,713	0,305	0,640
ART1	II	1,035	1,165	0,968	0,729	4,036	1,585	0,427	0,589
ART1	II	1,044	1,361	1,100	0,899	3,945	2,845	0,381	0,380
ART1	II	1,071	1,247	1,055	0,830	2,885	1,514	0,227	0,326
ART1	II	0,886	0,877	0,800	1,018	1,545	1,627	0,216	0,621
ART1	II	0,946	1,232	0,777	0,629	3,769	1,514	0,575	0,665
ART1	II	1,139	1,314	0,805	1,027	2,977	1,467	0,241	0,329
ART1	II	0,952	1,374	1,227	0,795	2,780	1,737	0,411	0,185
ART1	II	1,009	1,004	0,975	1,091	3,084	1,783	0,231	0,481
ART1	II	1,066	1,469	0,911	0,742	3,134	2,122	0,326	0,401
ART1	II	1,036	1,284	0,875	0,827	2,748	1,651	0,460	0,417
ART1	II	1,055	1,624	1,006	1,118	4,028	1,866	0,238	0,389
ART1	II	1,094	1,080	1,169	0,849	3,084	1,583	0,216	0,485
ART1	II	1,141	0,979	0,627	0,723	2,722	1,566	0,575	0,282
ART1	II	1,003	1,096	1,249	0,889	1,651	1,040	0,383	0,346
ART1	II	1,010	1,152	0,912	0,803	4,290	1,323	0,475	0,394
ART1	II	1,037	1,089	0,967	0,868	2,813	1,098	0,364	0,564
ART1	II	1,062	1,328	1,054	1,419	4,938	1,651	0,281	0,440
ART1	II	1,043	1,198	0,780	0,947	1,709	1,075	0,309	0,395
ART1	II	1,037	1,156	0,917	1,100	2,614	1,465	0,423	0,271
ART1	II	1,002	1,244	0,696	0,703	2,942	1,064	0,525	0,736
ART1	II	1,184	1,333	0,545	1,195	4,345	1,525	0,318	0,494
ART2	I	1,113	1,215	1,785	0,926	0,368	0,618	0,607	0,659
ART2	I	1,146	1,249	1,395	0,764	0,964	0,862	0,753	0,909
ART2	I	1,171	0,960	1,152	0,817	1,321	1,262	0,361	0,348
ART2	I	1,327	0,938	1,258	0,879	1,572	1,105	0,312	0,373
ART2	I	1,166	1,140	0,230	0,988	1,697	0,976	0,949	1,203
ART2	I	0,969	1,172	1,221	0,869	0,568	0,945	0,673	1,085
ART2	I	0,984	0,898	1,198	0,774	1,405	1,831	1,186	0,447
ART2	I	1,004	1,063	1,328	1,149	0,927	0,770	0,267	2,284
ART2	I	0,993	0,795	0,970	1,142	1,348	1,179	0,598	1,649
ART2	I	1,007	0,849	0,559	0,790	0,974	0,614	0,815	1,490
ART2	I	0,942	0,990	0,885	0,817	0,749	0,934	0,564	1,649
ART2	I	0,789	0,966	0,993	0,999	0,985	0,712	0,555	2,193
ART2	I	0,784	0,780	0,507	1,049	1,792	1,254	0,489	0,938
ART2	I	1,038	0,952	0,815	1,101	0,701	0,823	0,815	0,573
ART2	I	0,916	0,950	1,098	0,834	1,104	0,930	0,714	1,925
ART2	I	1,000	0,982	1,268	0,869	0,872	0,926	0,491	0,494
ART2	I	1,088	0,800	1,260	0,972	2,216	0,984	0,977	0,641
ART2	I	0,908	0,882	0,443	0,937	1,692	1,365	0,663	1,281
ART2	I	0,927	0,877	1,060	0,766	0,709	0,717	0,781	0,881
ART2	I	1,017	0,890	1,007	1,060	1,370	0,710	0,603	1,181
ART2	I	1,031	1,044	0,893	0,836	0,542	0,854	1,022	1,090
ART2	I	0,944	0,871	1,424	1,238	1,484	0,808	0,272	0,682
ART2	I	1,000	1,483	0,971	0,913	0,281	0,847	2,031	1,219
ART2	I	0,955	1,272	0,816	1,109	1,269	0,936	0,746	1,252
ART2	I	0,902	0,727	0,954	0,920	1,782	1,128	0,608	1,312
ART2	I	0,889	0,778	1,058	0,820	0,944	0,806	0,728	0,592
ART2	I	1,012	1,112	1,388	0,796	0,853	0,882	0,994	0,998
ART2	I	0,977	0,773	0,921	1,226	2,812	1,317	0,741	1,166
ART2	I	0,976	0,878	1,277	0,568	1,938	1,281	0,774	1,083

ART2	I	0,996	0,930	1,181	1,123	0,763	0,833	0,783	0,455
ART2	I	0,914	0,773	0,694	0,775	0,383	0,661	0,649	1,247
ART2	I	1,089	1,252	1,509	1,319	0,838	0,963	0,810	0,709
ART2	I	1,025	1,192	0,946	0,716	1,808	0,854	0,803	0,721
ART2	II	1,027	0,984	1,008	0,804	1,793	1,260	0,752	0,895
ART2	II	0,869	0,678	0,908	0,808	1,586	1,300	0,834	0,746
ART2	II	1,048	0,892	0,991	0,634	1,842	1,677	0,723	1,588
ART2	II	0,805	0,636	1,201	0,798	1,423	0,751	0,720	0,937
ART2	III	1,278	0,966	1,517	0,850	0,712	0,921	0,282	0,884
ART2	III	1,017	0,719	0,681	0,663	1,117	0,490	1,071	1,438
ART2	IV	1,099	1,042	1,188	1,004	1,206	0,944	0,989	0,528
ART2	IV	0,951	1,012	1,131	0,907	0,891	1,534	0,988	0,448
ART2	IV	1,009	1,213	0,827	0,956	1,312	1,444	0,656	0,514
ART2	IV	0,954	0,859	1,029	0,814	1,122	0,885	0,784	0,937
ART2	IV	0,918	0,892	1,181	0,914	0,880	0,945	0,707	0,430
ART2	IV	0,932	0,979	1,287	0,775	1,689	1,133	0,829	1,046
ART2	IV	0,984	0,786	1,269	1,084	1,478	1,254	0,525	1,286
ART2	IV	1,004	0,847	1,017	0,863	0,557	0,831	0,612	1,052
ART2	IV	1,000	1,123	1,019	0,826	1,385	0,844	0,746	1,065
ART3	I	1,023	0,962	0,929	0,888	1,959	1,120	0,782	0,738
ART3	I	1,013	0,623	1,059	1,255	2,420	1,489	0,699	0,358
ART3	I	0,995	1,005	1,098	0,816	0,857	1,832	0,645	1,785
ART3	I	0,895	1,085	1,255	1,609	0,243	1,072	0,790	1,567
ART3	I	0,935	0,952	1,138	0,707	1,944	1,649	0,635	0,704
ART3	I	0,973	0,846	0,984	0,860	1,521	1,001	0,576	0,870
ART3	I	0,912	1,025	0,757	1,134	2,041	1,458	0,557	0,625
ART3	I	0,952	0,983	0,789	0,782	1,628	1,056	0,537	0,909
ART3	I	0,919	0,916	0,799	0,827	2,145	1,033	0,810	0,781
ART3	I	1,017	0,968	1,277	0,899	2,590	1,216	0,714	0,292
ART3	I	1,004	1,034	0,904	0,753	1,899	1,061	0,674	0,464
ART3	I	0,920	0,909	0,974	0,849	2,601	1,217	0,570	0,595
ART3	I	0,949	0,999	0,887	0,867	2,558	1,303	0,623	0,392
ART3	I	0,959	0,805	0,843	0,569	3,834	1,437	0,956	0,566
ART3	I	1,053	1,018	0,994	0,901	2,136	1,081	0,941	0,341
ART3	I	1,011	0,800	1,184	0,992	1,877	1,200	0,701	0,459
ART3	II	0,893	0,907	1,016	0,731	2,495	1,005	0,683	0,233
ART3	II	1,075	0,808	1,443	0,942	2,403	1,947	0,698	0,309
ART3	II	0,899	0,956	1,142	0,858	2,252	1,558	0,811	0,819
ART3	II	0,875	0,763	0,942	0,960	2,968	1,656	0,785	0,588
ART3	II	1,054	0,986	1,160	0,985	2,768	1,533	0,660	0,634
ART3	II	1,042	0,910	0,804	0,815	2,589	1,296	1,136	0,692
ART3	II	0,919	0,827	0,796	1,024	1,727	1,040	0,739	0,792
ART3	II	0,921	0,753	0,696	0,754	2,261	1,683	0,628	0,804
ART3	II	0,961	0,948	1,356	0,740	2,893	1,124	0,607	0,375
ART3	II	0,924	1,053	1,678	1,105	2,133	1,066	0,285	0,425
ART3	II	1,125	1,043	0,905	0,917	2,413	1,465	1,305	0,616
ART3	II	1,025	0,910	1,245	0,975	2,699	1,385	0,388	0,622
ART3	II	0,991	1,054	1,655	1,138	2,458	1,787	0,355	0,519
ART3	II	0,923	0,887	1,114	0,832	1,861	1,167	0,950	0,616
ART3	II	0,983	1,030	1,237	0,993	2,875	1,424	0,899	0,693
ART3	II	0,901	1,069	1,152	0,873	4,534	1,144	0,577	0,719
ART3	III	0,950	1,254	1,177	0,860	2,486	1,153	0,816	0,313
ART3	III	1,010	0,731	1,749	0,741	3,273	1,172	0,500	0,558
ART3	IV	1,050	1,030	1,247	1,108	2,413	1,998	0,577	0,859
ART3	IV	0,959	1,039	1,052	0,907	2,053	1,816	0,675	0,545
ART4	I	0,924	1,106	0,829	0,858	0,657	1,060	0,486	0,737
ART4	I	1,095	1,218	1,232	1,052	1,625	1,028	0,378	0,640
ART4	I	1,146	0,934	0,639	0,751	2,257	0,931	0,453	1,146
ART4	I	1,096	1,340	0,843	0,691	3,861	2,025	0,890	0,595
ART4	I	1,045	1,272	0,937	0,869	1,863	1,162	0,543	0,393
ART4	II	1,336	1,123	1,248	0,878	2,151	1,399	0,239	0,433
ART4	II	1,043	0,985	1,035	0,813	2,756	1,058	0,269	0,773
ART4	II	1,619	1,158	1,135	0,837	2,194	0,876	0,695	0,445
ART4	II	1,326	1,050	1,242	0,831	2,594	1,344	0,727	0,243
ART4	II	1,041	1,039	0,789	0,802	1,007	0,699	0,657	0,507
ART4	II	1,147	1,166	1,595	0,842	1,722	1,957	0,749	0,718
ART4	II	1,020	0,854	1,221	1,044	1,741	0,746	0,423	0,589
ART4	II	0,979	0,841	0,959	0,957	2,124	1,620	0,514	0,642
ART4	II	0,985	1,163	1,138	0,821	3,169	1,209	0,492	0,206
ART4	III	1,025	0,959	1,031	0,849	1,832	1,007	0,392	0,491
ART4	III	1,158	0,963	1,146	0,973	2,097	1,401	0,744	0,338
ART4	III	1,024	0,914	0,919	0,815	2,864	1,575	0,587	0,498
ART4	IV	1,106	1,158	1,044	0,970	1,643	0,816	0,864	0,598
ART4	IV	0,966	0,893	1,521	0,806	1,104	0,909	0,684	0,537
ART4	IV	1,157	1,044	1,466	0,803	0,694	0,661	0,466	0,310
ART4	V	0,982	1,035	1,583	1,007	1,424	1,521	0,274	0,529
ART4	V	0,954	0,930	1,359	0,860	1,490	1,493	0,288	0,825
ART4	V	1,031	1,254	2,078	0,857	2,209	0,628	0,472	0,415
ART4	V	0,957	1,098	1,029	0,840	1,066	0,891	0,516	0,665
ART4	VI	1,165	1,201	0,980	0,879	0,863	0,833	0,437	0,595
ART4	VI	1,256	1,145	1,230	0,989	1,039	0,749	0,493	0,687
ART4	VI	1,052	1,491	0,821	0,984	0,946	1,282	0,643	0,555

ART4	VI	1,016	1,238	1,127	0,752	0,946	0,840	0,435	0,620
ART5	I	0,968	1,234	1,047	0,732	2,416	1,213	0,333	0,448
ART5	I	0,965	1,157	0,826	0,903	3,337	1,126	0,505	0,619
ART5	I	1,068	1,372	1,400	0,740	2,734	1,277	0,448	0,399
ART5	I	1,048	0,609	1,496	1,127	1,154	0,731	0,360	0,395
ART5	II	0,991	0,829	0,767	0,971	1,004	1,046	0,458	0,957
ART5	II	1,048	0,609	1,496	1,127	1,154	0,731	0,360	0,395
ART5	II	1,085	1,070	0,940	1,132	1,698	1,186	0,359	0,656
ART5	III	1,286	1,244	1,143	0,689	1,899	1,206	0,225	1,221
ART5	III	1,491	1,222	0,942	0,727	2,018	1,255	0,491	0,835
ART5	IV	1,010	1,029	1,286	0,641	1,497	0,724	0,519	0,560
ART5	IV	0,951	1,111	0,847	0,748	1,646	1,303	0,361	0,674
ART5	IV	1,243	1,040	0,950	0,773	3,229	1,498	0,518	0,699
ART5	IV	0,975	1,686	0,666	0,525	1,751	1,111	0,632	0,559
ART5	IV	0,979	1,592	0,856	0,797	0,774	1,254	0,683	0,349
ART5	IV	1,095	1,080	1,612	0,829	2,025	1,395	0,236	0,488
ART5	IV	1,055	1,304	1,194	0,662	1,891	1,406	0,497	0,558
ART5	IV	1,061	1,131	1,133	0,686	1,103	0,819	0,281	0,443
ART5	IV	0,953	1,261	1,187	0,743	2,933	1,136	0,454	0,656
ART5	IV	0,967	1,225	0,877	0,635	1,020	1,232	0,593	0,569
ART5	IV	1,009	1,760	0,948	0,993	2,236	1,191	0,755	0,690
ART5	IV	1,000	1,906	1,117	0,825	2,557	1,030	0,412	0,490
ART5	IV	1,084	0,991	1,423	0,692	2,526	0,820	0,669	0,259
ART5	IV	1,052	1,389	1,275	0,682	2,583	1,004	0,470	0,428
ART5	IV	1,070	1,461	0,930	0,650	2,025	0,995	0,492	0,580
ART5	IV	1,130	1,041	1,272	0,869	2,017	1,546	0,484	0,398
ART5	IV	1,104	1,324	1,327	0,663	2,117	1,397	0,584	0,532
ART5	IV	0,949	1,408	1,112	0,801	3,711	1,556	0,426	0,823
ART5	IV	1,025	0,977	1,102	1,035	0,917	1,111	0,406	0,679
ART5	IV	1,003	1,368	1,131	0,861	2,625	1,659	0,395	0,443
ART5	IV	1,037	1,401	1,020	0,679	2,397	1,145	0,547	0,594
ART5	IV	0,954	1,158	0,997	0,754	2,317	0,896	0,429	0,527
ART5	IV	1,285	1,496	1,804	0,725	3,045	1,544	0,583	0,458
ART5	IV	1,087	1,051	1,258	0,678	0,871	0,664	0,496	0,433
ART5	IV	1,026	1,010	1,936	0,741	3,491	0,771	0,448	0,454
ART5	IV	1,269	1,043	0,764	0,550	2,433	1,400	0,373	0,665
ART5	IV	1,040	1,352	0,847	0,855	1,086	1,220	0,532	0,579
ART5	IV	1,168	0,923	0,947	0,712	1,424	1,444	0,479	0,535
ART5	IV	1,361	1,064	1,150	0,632	1,429	0,923	0,417	0,328
ART5	IV	1,745	1,181	1,199	0,996	2,351	1,000	0,345	0,378
ART5	IV	1,023	1,439	0,730	0,643	2,044	1,242	0,328	0,582
ART5	IV	0,982	1,516	1,119	0,616	2,164	1,109	0,647	0,519
ART5	V	1,052	1,258	1,158	0,845	3,743	1,395	0,568	0,941
ART5	V	1,045	0,707	0,992	0,926	3,008	2,378	0,500	0,546
ART5	V	0,905	1,095	1,237	0,666	3,084	1,360	0,428	0,548
ART6	I	1,123	1,101	1,537	0,896	1,729	0,854	0,569	0,631
ART6	I	0,990	0,988	0,855	0,824	1,766	0,919	0,611	0,947
ART6	I	0,964	1,027	0,843	0,795	1,396	0,915	0,419	1,117
ART6	II	0,950	0,950	1,138	0,787	8,646	2,315	0,839	0,468
ART6	II	0,992	0,990	0,877	1,096	3,201	1,496	0,262	1,589
ART6	II	1,005	0,801	1,398	0,760	6,152	2,616	0,444	0,808
ART6	II	1,069	0,880	1,089	0,984	2,873	1,384	0,400	0,883
ART6	III	1,024	1,339	0,852	1,220	0,428	0,957	0,536	1,935
ART6	III	1,020	1,463	1,675	0,980	0,443	0,820	0,451	0,588
ART6	III	0,944	1,736	0,871	2,729	0,351	0,813	0,748	0,877
ART6	III	1,050	0,925	1,106	1,560	0,368	1,150	0,440	0,739
ART6	IV	1,050	1,615	0,711	1,440	3,745	0,991	2,338	2,285
ART6	IV	0,993	1,134	1,436	0,908	5,060	1,171	0,495	0,449
ART6	IV	1,041	1,014	0,876	0,774	4,407	1,304	0,345	1,541
ART6	IV	0,973	0,913	0,969	0,883	3,926	1,657	0,427	1,019
ART6	V	1,000	1,470	1,109	0,767	2,271	1,039	0,458	0,618
ART6	V	0,986	1,281	1,035	0,859	1,965	1,227	0,494	0,424
ART6	V	0,972	1,282	1,148	0,638	2,695	1,813	0,318	1,039
ART6	V	1,007	1,110	0,888	0,967	2,172	1,687	0,429	1,126
ART6	V	0,959	1,195	1,068	0,834	1,539	1,171	0,418	1,075
ART6	V	0,930	0,965	1,183	0,830	2,165	0,900	0,268	1,119
ART6	V	1,010	1,008	1,029	0,670	2,038	1,182	0,374	1,224
ART6	V	0,996	1,119	0,835	0,851	1,161	1,009	0,460	0,819
ART6	V	1,025	0,892	1,083	0,753	0,749	1,371	0,350	1,231
ART6	V	0,972	0,838	0,834	0,727	3,278	1,763	0,461	1,091
ART6	V	0,959	1,147	1,065	0,778	1,870	0,986	0,472	1,290
ART6	VI	0,978	0,864	0,933	0,910	4,927	1,682	0,374	1,346
ART6	VI	0,935	0,713	1,655	0,555	2,544	1,021	0,250	0,990
ART6	VI	1,047	1,017	2,372	0,740	4,444	1,818	0,146	1,125
ART6	VII	0,991	1,248	0,952	0,812	0,520	0,717	0,742	0,981
ART6	VII	0,990	1,607	1,387	0,879	1,865	0,936	0,476	0,647
ART6	VII	1,028	1,292	1,087	0,823	1,133	0,719	0,431	0,495
ART6	VII	1,054	1,130	0,925	0,811	1,080	0,808	0,274	1,201
ART6	VII	0,995	1,248	0,911	1,279	2,242	1,357	0,568	0,714
ART6	VII	0,963	1,129	0,803	0,903	0,367	0,875	0,475	1,225

Figure 4c

	# of proviral sequence						
	PD-1+	TIGIT+	HLA-DR+	ICOS+	α4+β1+	Tcm	Tem/Ttm
Unique	29	15	10	13	52	27	37
Clonal	103	96	56	31	185	51	167

Figure 5c

ART1		ART2		ART3		ART4		ART5		ART6	
Sequenc length (pb)		Sequenc length (pb)		Sequenc length (pb)		Sequenc length (pb)		Sequenc length (pb)		Sequenc length (pb)	
p24+	p24-	p24+	p24-	p24+	p24-	p24+	p24-	p24+	p24-	p24+	p24-
9087	1182	9098	3460	9007	3875	9057	4950	9017	3643	9023	4437
9087	3141	9098	5905	9039	2357	8930	8511	9017	8983	8945	3326
9087	1513	8866	2273	9181	3875	8206	9013	9006	6433	9045	4060
9087	4231	8625	5905	9039	1360	9062	8976	9053	1717	9023	4437
9055	1903	9098	5905	9039	2854	9057	2982	9027	8983	9094	4804
9055	1364	8852	5905	9039	8515	9057	9013	9028	8969	8775	2075
9087	9006	8625	5905	9040	3875	8866	8895	9029	8525	9064	5467
9087	4276	8625	5905	9039	4111	9057	4870	9047	8888	9071	8989
9087	3450	8625	5905	9039	2745	9062	5730	9027	4346	8895	4804
9087	4521	8625	1479	9039	1800	9062	8662	9020	8983	9071	4120
9087	1099	8625	5905	9039	8981	8560	2983	9030	8983	8880	2992
9087	1956	8625	5905	9007	8958	8560	8180	9017	4987	9094	2449
9087	4145	8853	5905	9039	2468	8867	8511	9028	4346	8796	4062
9067	1048	8625	1832	9039	4941	8866	3043	8586	8992	9094	9010
8997	2068	8630	1299	9039	5113	8560	9008	9025	8983	9094	8930
9066	2235	8625	5905	9052	4768	8866	8157	8993	3643	8880	4804
9066	3439	8866	5905	9040	2865	8866	7860	9029	8962	8880	1274
9066	1496	8876	5905	9039	4719	8866	2676	9028	2179	9089	8964
9055	4495	8637	5905	9213	7427	8990	4950	9028	4513	9045	1897
9067	1513	8625	5905	9039	3055	8866	5730	9026	8969	8764	4807
9066	3939	8627	5905	9039	3055	8560	8941	9030	9010	9033	4781
9065	3939	9100	5905	8651	3264	8560	2982	9017	5089	9014	4642
9062	4521	9120	5905	9039	2290	8866	4378	8947	6433	9023	8869
9064	3939	8631	5905	9017	2849	8662	5154	9030	8969	8774	4598
9066	2641	9095	5467	9020	4264	8941	5154	9026		9070	4062
9021	1119	8635	5905	9017	2481	8511	5193	9029		8772	6648
9017	1016	9097	5905	9016	3875	9008	9013	9043		9061	4804
9065	4145	8625	8582	8497	1028	9061	9013	9028		9006	4298
9066	961	8595	5905	9020	6977	9057	8511	9021		9024	1012
9067	4145	8624	5905	9192	2143	8866	9008	9050		8735	9007
9055	1756	8631	5905	9192	7686	9034	7860	8606		9062	4804
8996	1903	9104	5905	9193	4941	8915	9008	9050		9060	4804
9056	1119	8626	5905	9019	9034	8874	8937	9050		9042	4307
9069	9016	8852	5905	9017	4264	9062	529	9050		9036	9028
9067	988	9098	5905	8986	2622	8995	2982	9064		8869	4542
9056	8937	8638	5905	9019	2941	8772	8957	8606		8837	3407
9064	6212	8625	5905	9018	9004	8866	8157	9050		9032	2338
8999	2235	8636	5905	9013	3302	8592	8294	9050		8902	8996
9064	1496	8436	6316	9019	5368	8898	7860	8606		8743	5190
9063	3939	9098	3167	8789	2463	8898	8817	9050		8742	8382
9068	1099	8634	5905	9019	4941	8898	6632	9050		9024	4804
9066	1091	8635	5905	9109	3875		8176	9050		8964	4804
9055	1970	8638	5905	9128	3302		6632	8606		8847	4307
9066	1182	8636	5905	9019	4877		5154	9041		9032	4060
9062	1540	8632	8638	9017	4264		5154	9041		9033	4804
9055	4871	8860	5905	9006	4210		6632	9050		9033	4704
9064	4401	8624	5905	9019	4264		1156	9050		9017	7974
9068	3939	8625	5905	9017	3131		4265	9050		9036	4804
9063	2125	9098	5905	9020	9062		4950	9050		9004	8956
8995	7175	8657	5905	9192	8515		8941	9050		8770	8930
9066	1472	8657	5905		3554		8985	9050		9027	4141
8994	2600	8884	5905		4264			9050		8774	4062
	4276	8657	5905		8515					9027	4062
	4145	8657	5905							9005	2362
	4175	8657								9013	5199
	1394									9042	757
	4175									9030	4062
	1182									9007	4804
	3939										3826
	9016										4804
	6178										3629
	4521										5467
	8970										1747
	1182										1708
	8937										8906
	941										8986
	4231										8998
	6178										8799
											4804
											3555
											3326
											4804
											4804
											4761
											4804
											5337

Figure 5d

	p24+	p24-
Proviral integrity	# of proviral sequence	
Intact	12	7
Inversion	0	9
Hypermutations	0	26
Large deletion	64	253
Stop codons	43	9
Ψ defects	189	22

Figure 5e

	p24+ # of proviral sequence		p24+ # of proviral sequence		p24+ # of proviral sequence		p24+ # of proviral sequence		p24+ # of proviral sequence		p24+ # of proviral sequence	
	ART1	ART1	ART2	ART2	ART3	ART3	ART4	ART4	ART5	ART5	ART6	ART6
Total seq	52	68	55	54	50	53	41	51	52	24	58	76
Psi (# intact)	0	56	0	6	7	41	5	23	4	16	12	64
Gag (# intact)	48	34	10	4	9	29	12	23	46	17	19	17
Pol (# intact)	48	2	11	0	46	2	12	14	46	12	40	26
Vif (# intact)	52	5	55	47	50	6	41	32	52	17	58	15
Vpr (# intact)	52	5	55	3	50	7	41	32	52	16	58	15
Tat (# intact)	52	7	55	47	50	5	41	32	52	16	58	17
Rev (# intact)	52	5	55	47	50	8	41	31	52	16	58	14
Vpu (# intact)	52	5	55	47	50	6	41	33	52	16	58	15
Nef (# intact)	52	10	0	44	38	1	41	31	45	16	56	18
Env (# intact)	52	5	54	46	49	5	41	31	51	16	56	12
RRE (# intact)	52	11	55	47	50	15	41	35	52	16	58	19

Figure 6a

PD-1 (FI/StimPop_MFI)				TIGIT (FI/StimPop_MFI)				HLA-DR (FI/StimPop_MFI)				ICOS (FI/StimPop_MFI)			
Large deletion	Stop codon	Ψ defects	Intact	Large deletion	Stop codon	Ψ defects	Intact	Large deletion	Stop codon	Ψ defects	Intact	Large deletion	Stop codon	Ψ defects	Intact
1,113	1,278	1,974	1,050	1,215	0,966	1,375	1,030	1,785	1,517	1,523	1,247	0,926	0,850	1,390	1,108
1,146	1,027	1,343	0,959	1,249	0,984	1,227	1,039	1,395	1,008	1,007	1,052	0,764	0,804	0,826	0,907
1,171	0,869	1,428	0,950	0,960	0,678	1,257	1,254	1,152	0,908	1,124	1,177	0,817	0,808	0,963	0,860
1,327	1,017	1,283	1,038	0,938	0,719	1,521	1,017	1,258	0,681	1,150	0,875	0,879	0,663	0,744	0,854
1,166	1,022	1,437	1,010	1,140	0,886	1,376	0,731	0,230	0,920	1,189	1,749	0,988	1,010	0,965	0,741
0,969	1,038	1,365	0,988	1,172	0,952	1,427	0,853	1,221	0,815	1,524	0,911	0,869	1,101	0,830	0,932
0,984	1,048	1,408	1,203	0,898	0,892	1,420	0,651	1,198	0,991	0,723	1,067	0,774	0,634	1,107	0,931
1,004	0,805	1,706	1,041	1,063	0,636	1,539	1,069	1,328	1,201	1,091	1,126	1,149	0,798	1,191	1,181
0,993	1,119	1,672	0,978	0,795	0,876	1,506	0,864	0,970	0,990	0,690	0,933	1,142	1,051	0,926	0,910
1,007	1,044	1,572	0,935	0,849	0,912	1,400	0,713	0,559	1,067	1,148	1,655	0,790	0,949	0,753	0,555
0,942	0,965	1,245	1,047	0,990	1,014	1,148	1,017	0,885	1,100	1,369	2,372	0,817	0,793	1,008	0,740
0,789	1,011	1,081		0,966	0,870	1,293		0,993	0,882	1,384		0,999	0,896	0,776	
0,784	1,000	1,195		0,780	1,470	1,189		0,507	1,109	1,127		1,049	0,767	0,914	
1,038	1,050	0,942		0,952	1,615	0,909		0,815	0,711	0,808		1,101	1,440	1,011	
0,916	0,986	0,953		0,950	1,281	0,646		1,098	1,035	1,280		0,834	0,859	1,001	
1,000	0,980	1,041		0,982	0,824	1,036		1,268	0,958	1,159		0,869	1,409	1,127	
1,088	1,024	0,919		0,800	1,339	1,449		1,260	0,852	0,873		0,972	1,220	0,927	
0,908	1,021	1,179		0,882	0,947	0,804		0,443	1,107	1,309		0,937	0,892	1,296	
0,927	1,020	1,019		0,877	1,463	1,261		1,060	1,675	0,493		0,766	0,980	1,403	
1,017	0,950	0,938		0,890	1,272	0,822		1,007	0,996	1,044		1,060	1,365	1,076	
1,031	0,944	1,043		1,044	1,736	1,242		0,893	0,871	1,014		0,836	2,729	1,001	
0,944	0,983	0,983		0,871	0,780	0,953		1,424	1,005	0,996		1,238	1,057	0,810	
0,938	0,993	0,980		0,867	1,134	1,224		0,883	1,436	0,815		0,935	0,908	0,880	
1,000	0,972	0,984		1,483	1,282	1,262		0,971	1,148	0,571		0,913	0,638	0,752	
0,955	1,041	1,069		1,272	1,014	1,665		0,816	0,876	0,730		1,109	0,774	0,897	
0,902	1,007	0,980		0,727	1,110	1,132		0,954	0,888	0,929		0,920	0,967	1,277	
0,889	1,048	1,022		0,778	1,118	1,021		1,058	0,916	1,134		0,820	0,835	0,959	
1,012	0,964	1,035		1,112	1,083	1,165		1,388	0,966	0,968		0,796	1,285	0,729	
0,977	0,959	1,044		0,773	1,195	1,361		0,921	1,068	1,100		1,226	0,834	0,899	
0,976	1,074	1,071		0,878	1,247	1,247		1,277	1,068	1,055		0,568	0,824	0,830	
0,996	0,998	0,886		0,930	1,022	0,877		1,181	0,617	0,800		1,123	0,604	1,018	
0,914	1,051	1,061		0,773	0,976	1,029		0,694	0,789	0,706		0,775	0,765	0,909	
1,005	0,930	0,946		1,178	0,965	1,232		1,329	1,183	0,777		1,207	0,830	0,629	
1,089	1,050	1,139		1,252	0,925	1,314		1,509	1,106	0,805		1,319	1,560	1,027	
1,025	1,010	0,952		1,192	1,008	1,374		0,946	1,029	1,227		0,716	0,670	0,795	
0,868	0,996	1,009		1,026	1,119	1,004		0,885	0,835	0,975		1,081	0,851	1,091	
0,940	1,025	1,066		1,051	0,892	1,469		0,739	1,083	0,911		0,973	0,753	0,742	
0,994	0,972	0,998		0,886	0,838	1,013		0,875	0,834	1,133		1,337	0,727	0,826	
1,077	0,959	1,036		1,348	1,147	1,284		1,184	1,065	0,875		1,213	0,778	0,827	
1,040	0,952	1,055		0,869	0,878	1,624		1,069	0,817	1,006		0,828	0,641	1,118	
0,924	0,973	1,094		1,106	0,913	1,080		0,829	0,969	1,169		0,858	0,883	0,849	
1,095	0,983	1,141		1,218	1,034	0,979		1,232	1,456	0,627		1,052	0,879	0,723	
1,146		1,003		0,934		1,096		0,639		1,249		0,751		0,889	
1,096		1,010		1,340		1,152		0,843		0,912		0,691		0,803	
0,952		1,037		1,007		1,089		1,091		0,967		0,818		0,868	
1,045		1,062		1,272		1,328		0,937		1,054		0,869		1,419	
1,226		1,043		1,320		1,198		1,186		0,780		1,077		0,947	
1,026		1,037		1,028		1,156		0,876		0,917		0,770		1,100	
0,968		1,002		1,234		1,244		1,047		0,696		0,732		0,703	
0,965		1,039		1,157		1,145		0,826		0,807		0,903		1,312	
1,068		1,184		1,372		1,333		1,400		0,545		0,740		1,195	
0,926		1,018		1,051		1,029		2,299		1,024		0,785		0,715	
1,048		1,099		0,609		1,042		1,496		1,188		1,127		1,004	
1,123		0,951		1,101		1,012		1,537		1,131		0,896		0,907	
0,950		1,009		0,950		1,213		1,138		0,827		0,787		0,956	
0,947		0,954		0,884		0,859		0,880		1,029		0,889		0,814	
0,992		1,006		0,990		0,948		0,877		1,274		1,096		0,922	
1,005		0,918		0,801		0,892		1,398		1,181		0,760		0,914	
0,995		0,932		1,248		0,979		0,911		1,287		1,279		0,775	
0,990		0,984		0,988		0,786		0,855		1,269		0,824		1,084	
0,964		1,004		1,027		0,847		0,843		1,017		0,795		0,863	
1,065		1,000		1,113		1,123		0,576		1,019		1,008		0,826	
1,069		0,893		0,880		0,907		1,089		1,016		0,984		0,731	
		1,075				0,808				1,443				0,942	
		0,899				0,956				1,142				0,858	
		0,875				0,763				0,942				0,960	
		1,054				0,986				1,160				0,985	
		1,042				0,910				0,804				0,815	
		0,919				0,827				0,796				1,024	
		0,921				0,753				0,696				0,754	
		0,961				0,948				1,356				0,740	
		0,924				1,053				1,678				1,105	
		1,125				1,043				0,905				0,917	
		0,980				0,929				0,801				0,870	
		1,025				0,910				1,245				0,975	
		0,991				1,054				1,655				1,138	
		0,950				0,962				1,042				1,005	
		1,076				1,241				1,101				0,902	
		0,923				0,887				1,114				0,832	
		0,983				1,030				1,237				0,993	
		0,901				1,069				1,152				0,873	
		1,023				0,962				0,929				0,888	
		1,013				0,623				1,059				1,255	
		0,995				1,005				1,098				0,816	
		0,895				1,085				1,255				1,609	
		0,935				0,952				1,138				0,707	
		0,953				0,796				1,079				1,022	
		0,973				0,846				0,984				0,860	
		0,912				1,025				0,757				1,134	
		0,952				0,983				0,789				0,782	
		0,919				0,916				0,799				0,827	
		1,017				0,968				1,277				0,899	

1,015	1,071	0,714	1,207
0,923	0,729	0,770	0,967
1,004	1,034	0,904	0,753
0,920	0,909	0,974	0,849
0,949	0,999	0,887	0,867
0,959	0,805	0,843	0,569
1,053	1,018	0,994	0,901
1,011	0,800	1,184	0,992
0,944	0,925	1,016	0,884
0,959	0,793	1,140	0,856
1,076	0,824	0,889	0,880
1,025	0,961	1,008	0,899
0,982	1,035	1,583	1,007
1,598	0,921	0,978	0,928
0,954	0,930	1,359	0,860
1,165	1,201	0,980	0,879
1,256	1,145	1,230	0,989
1,336	1,123	1,248	0,878
1,043	0,985	1,035	0,813
1,579	1,176	0,551	0,765
1,619	1,158	1,135	0,837
1,326	1,050	1,242	0,831
1,106	1,158	1,044	0,970
1,041	1,039	0,789	0,802
1,147	1,166	1,595	0,842
0,966	0,893	1,521	0,806
1,031	1,254	2,078	0,857
1,052	1,491	0,821	0,984
0,957	1,098	1,029	0,840
1,020	0,854	1,221	1,044
1,009	1,059	0,877	0,953
0,894	0,939	1,244	1,046
0,979	0,841	0,959	0,957
1,016	1,238	1,127	0,752
1,157	1,044	1,466	0,803
0,985	1,163	1,138	0,821
1,025	0,959	1,031	0,849
1,158	0,963	1,146	0,973
1,024	0,914	0,919	0,815
1,010	1,029	1,286	0,641
0,951	1,111	0,847	0,748
1,243	1,040	0,950	0,773
1,052	1,258	1,158	0,845
0,975	1,686	0,666	0,525
0,979	1,592	0,856	0,797
1,095	1,080	1,612	0,829
1,045	0,707	0,992	0,926
1,055	1,304	1,194	0,662
1,061	1,131	1,133	0,686
0,953	1,261	1,187	0,743
0,967	1,225	0,877	0,635
1,009	1,760	0,948	0,993
1,000	1,906	1,117	0,825
0,985	1,006	1,031	0,685
1,084	0,991	1,423	0,692
1,052	1,389	1,275	0,682
1,070	1,461	0,930	0,650
1,130	1,041	1,272	0,869
1,104	1,324	1,327	0,663
0,949	1,408	1,112	0,801
1,025	0,977	1,102	1,035
1,003	1,368	1,131	0,861
1,037	1,401	1,020	0,679
0,905	1,095	1,237	0,666
0,954	1,158	0,997	0,754
0,991	0,829	0,767	0,971
1,285	1,496	1,804	0,725
1,087	1,051	1,258	0,678
1,026	1,010	1,936	0,741
1,269	1,043	0,764	0,550
0,957	1,342	1,074	0,692
1,131	1,396	1,879	0,713
1,286	1,244	1,143	0,689
1,040	1,352	0,847	0,855
1,168	0,923	0,947	0,712
1,048	0,609	1,496	1,127
1,085	1,070	0,940	1,132
1,361	1,064	1,150	0,632
1,745	1,181	1,199	0,996
1,491	1,222	0,942	0,727
1,023	1,439	0,730	0,643
1,407	1,022	1,030	0,715
1,301	0,863	1,769	0,722
0,982	1,516	1,119	0,616
1,056	0,983	1,018	0,897
0,991	1,248	0,952	0,812
0,990	1,607	1,387	0,879
1,028	1,292	1,087	0,823
1,054	1,130	0,925	0,811
0,995	1,248	0,911	1,279
0,963	1,129	0,803	0,903
1,053	0,987	0,837	0,846
0,954	1,174	1,005	0,935
1,007	0,938	1,233	0,962
1,079	0,819	1,077	0,974
1,111	0,983	1,016	0,859

Figure 6a

α4 (FI/StimPop_MFI)				β1 (FI/StimPop_MFI)				CD45RA (FI/StimPop_MFI)				CCR7 (FI/StimPop_MFI)			
Large deletion	Stop codon	Ψ defects	Intact	Large deletion	Stop codon	Ψ defects	Intact	Large deletion	Stop codon	Ψ defects	Intact	Large deletion	Stop codon	Ψ defects	Intact
0,368	0,712	4,125	2,413	0,618	0,921	2,331	1,998	0,607	0,282	0,331	0,577	0,659	0,884	0,599	0,859
0,964	1,793	2,742	2,053	0,862	1,260	1,415	1,816	0,753	0,752	0,574	0,675	0,909	0,895	0,708	0,545
1,321	1,586	2,762	2,486	1,262	1,300	1,444	1,153	0,361	0,834	0,554	0,816	0,348	0,746	0,667	0,313
1,572	1,117	2,768	3,416	1,105	0,490	1,754	1,297	0,312	1,071	0,543	0,858	0,373	1,438	0,704	0,352
1,697	0,841	3,772	3,273	0,976	0,817	1,595	1,172	0,949	0,517	0,557	0,500	1,203	1,272	0,352	0,558
0,568	0,701	3,376	1,333	0,945	0,823	1,575	1,012	0,673	0,815	0,459	0,595	1,085	0,573	0,677	0,626
1,405	1,842	4,125	5,007	1,831	1,677	1,763	1,607	1,186	0,723	0,469	0,803	0,447	1,588	0,629	0,411
0,927	1,423	2,822	3,410	0,770	0,751	1,687	1,463	0,267	0,720	0,549	0,479	2,284	0,937	0,678	0,781
1,348	0,635	3,050	4,927	1,179	0,817	1,661	1,682	0,598	0,367	0,470	0,374	1,649	0,951	0,534	1,346
0,974	2,284	3,351	2,544	0,614	1,578	1,696	1,021	0,815	0,788	0,712	0,250	1,490	0,476	0,336	0,990
0,749	1,393	2,645	4,444	0,934	1,597	1,640	1,818	0,564	0,617	0,284	0,146	1,649	1,032	0,624	1,125
0,985	2,285	3,541		0,712	1,278	1,979		0,555	0,684	0,541		2,193	0,526	0,584	
1,792	2,271	3,157		1,254	1,039	1,499		0,489	0,458	0,608		0,938	0,618	0,520	
0,701	3,745	2,022		0,823	0,991	1,699		0,815	2,338	0,525		0,573	2,285	0,761	
1,104	1,965	1,231		0,930	1,227	1,190		0,714	0,494	1,261		1,925	0,424	0,329	
0,872	3,385	2,167		0,926	1,327	1,624		0,491	0,561	0,419		0,494	0,810	0,511	
2,216	0,428	4,161		0,984	0,957	1,968		0,977	0,536	0,406		0,641	1,935	0,571	
1,692	4,754	3,466		1,365	1,713	1,737		0,663	0,839	0,499		1,281	0,761	0,469	
0,709	0,443	3,116		0,717	0,820	1,689		0,781	0,451	0,880		0,881	0,588	0,556	
1,370	0,394	1,918		0,710	1,109	1,187		0,603	0,766	0,593		1,181	1,087	0,469	
0,542	0,351	2,241		0,854	0,813	2,010		1,022	0,748	0,443		1,090	0,877	0,489	
1,484	1,969	3,528		0,808	1,086	1,390		0,272	0,401	0,433		0,682	1,174	0,384	
1,385	5,060	3,841		0,993	1,171	2,089		0,726	0,495	0,554		0,554	0,449	0,488	
0,281	2,695	2,802		0,847	1,813	1,229		2,031	0,318	0,332		1,219	1,039	0,444	
1,269	4,407	4,174		0,936	1,304	1,713		0,746	0,345	0,305		1,252	1,541	0,640	
1,782	2,172	2,522		1,128	1,687	1,277		0,608	0,429	0,103		1,312	1,126	2,159	
0,944	3,032	2,753		0,806	0,907	1,332		0,728	0,280	0,437		0,592	0,972	0,362	
0,853	0,684	4,036		0,882	2,114	1,585		0,994	0,293	0,427		0,998	0,871	0,589	
2,812	1,539	3,945		1,317	1,171	2,845		0,741	0,418	0,381		1,166	1,075	0,380	
1,938	0,457	2,885		1,281	1,804	1,514		0,774	0,180	0,227		1,083	1,115	0,326	
0,763	2,929	1,545		0,833	1,553	1,627		0,783	0,476	0,216		0,455	0,855	0,621	
0,383	3,785	1,497		0,661	1,400	0,929		0,649	0,448	1,286		1,247	0,958	0,498	
1,686	2,165	3,769		0,704	0,900	1,514		0,822	0,268	0,575		0,260	1,119	0,665	
0,838	0,368	2,977		0,963	1,150	1,467		0,810	0,440	0,241		0,709	0,739	0,329	
1,808	2,038	2,780		0,854	1,182	1,737		0,803	0,374	0,411		0,721	1,224	0,185	
0,619	1,161	3,084		0,829	1,009	1,783		0,933	0,460	0,231		0,896	0,819	0,481	
3,083	0,749	3,134		1,431	1,371	2,122		0,595	0,350	0,326		0,706	1,231	0,401	
1,647	3,278	1,969		0,908	1,763	1,006		0,809	0,461	0,465		0,637	1,091	0,509	
0,556	1,870	2,748		1,003	0,986	1,651		0,457	0,472	0,460		0,607	1,290	0,417	
2,257	2,730	4,028		1,450	1,259	1,866		0,549	0,317	0,238		0,466	1,302	0,389	
0,657	3,926	3,084		1,060	1,657	1,583		0,486	0,427	0,216		0,737	1,019	0,485	
1,625	1,345	2,722		1,028	1,233	1,566		0,378	0,417	0,575		0,640	0,660	0,282	
2,257		1,651		0,931		1,040		0,453		0,383		1,146		0,346	
3,861		4,290		2,025		1,323		0,890		0,475		0,595		0,394	
2,690		2,813		1,438		1,098		0,523		0,364		0,537		0,564	
1,863		4,938		1,162		1,651		0,543		0,281		0,393		0,440	
1,464		1,709		0,973		1,075		0,453		0,309		0,281		0,395	
2,980		2,614		1,449		1,465		0,689		0,423		0,857		0,271	
2,416		2,942		1,213		1,064		0,333		0,525		0,448		0,736	
3,337		2,003		1,126		1,912		0,505		0,235		0,619		1,368	
2,734		4,345		1,277		1,525		0,448		0,318		0,399		0,494	
3,088		3,295		0,927		2,024		0,299		0,330		0,548		0,304	
1,154		1,206		0,731		0,944		0,360		0,989		0,395		0,528	
1,729		0,891		0,854		1,534		0,569		0,988		0,631		0,448	
8,646		1,312		2,315		1,444		0,839		0,656		0,468		0,514	
3,360		1,122		1,643		0,885		0,557		0,784		0,804		0,937	
3,201		0,620		1,496		1,541		0,262		0,561		1,589		0,631	
6,152		0,880		2,616		0,945		0,444		0,707		0,808		0,430	
2,242		1,689		1,357		1,133		0,568		0,829		0,714		1,046	
1,766		1,478		0,919		1,254		0,611		0,525		0,947		1,286	
1,396		0,557		0,915		0,831		0,419		0,612		1,117		1,052	
1,317		1,385		0,896		0,844		0,404		0,746		0,984		1,065	
2,873		2,495		1,384		1,005		0,400		0,683		0,883		0,233	
		2,403				1,947				0,698				0,309	
		2,252				1,558				0,811				0,819	
		2,968				1,656				0,785				0,588	
		2,768				1,533				0,660				0,634	
		2,589				1,296				1,136				0,692	
		1,727				1,040				0,739				0,792	
		2,261				1,683				0,628				0,804	
		2,893				1,124				0,607				0,375	
		2,133				1,066				0,285				0,425	
		2,413				1,465				1,305				0,616	
		2,851				1,547				0,926				0,647	
		2,699				1,385				0,388				0,622	
		2,458				1,787				0,355				0,519	
		2,302				1,637				0,657				0,747	
		2,838				1,471				0,959				0,364	
		1,861				1,167				0,950				0,616	
		2,875				1,424				0,899				0,693	
		4,534				1,144				0,577				0,719	
		1,959				1,120				0,782				0,738	
		2,420				1,489				0,699				0,358	
		0,857				1,832				0,645				1,785	
		0,243				1,072				0,790				1,567	
		1,944				1,649				0,635				0,704	
		2,486				1,567				0,492				0,460	
		1,521				1,001				0,576				0,870	
		2,041				1,458				0,557				0,625	
		1,628				1,056				0,537				0,909	
		2,145				1,033				0,810				0,781	
		2,590				1,216				0,714				0,292	

0,976	1,716	1,025	2,028
1,845	0,796	0,692	0,473
1,899	1,061	0,674	0,464
2,601	1,217	0,570	0,595
2,558	1,303	0,623	0,392
3,834	1,437	0,956	0,566
2,136	1,081	0,941	0,341
1,877	1,200	0,701	0,459
1,638	0,844	0,548	0,389
3,896	1,808	0,411	1,013
0,517	0,601	0,469	0,486
1,948	1,824	0,418	0,717
1,424	1,521	0,274	0,529
1,782	1,303	0,593	0,536
1,490	1,493	0,288	0,825
0,863	0,833	0,437	0,595
1,039	0,749	0,493	0,687
2,151	1,399	0,239	0,433
2,756	1,058	0,269	0,773
2,308	0,859	0,675	0,581
2,194	0,876	0,695	0,445
2,594	1,344	0,727	0,243
1,643	0,816	0,864	0,598
1,007	0,699	0,657	0,507
1,722	1,957	0,749	0,718
1,104	0,909	0,684	0,537
2,209	0,628	0,472	0,415
0,946	1,282	0,643	0,555
1,066	0,891	0,516	0,665
1,741	0,746	0,423	0,589
0,852	0,921	0,604	0,564
1,310	0,767	0,590	0,618
2,124	1,620	0,514	0,642
0,946	0,840	0,435	0,620
0,694	0,661	0,466	0,310
3,169	1,209	0,492	0,206
1,832	1,007	0,392	0,491
2,097	1,401	0,744	0,338
2,864	1,575	0,587	0,498
1,497	0,724	0,519	0,560
1,646	1,303	0,361	0,674
3,229	1,498	0,518	0,699
3,743	1,395	0,568	0,941
1,751	1,111	0,632	0,559
0,774	1,254	0,683	0,349
2,025	1,395	0,236	0,488
3,008	2,378	0,500	0,546
1,891	1,406	0,497	0,558
1,103	0,819	0,281	0,443
2,933	1,136	0,454	0,656
1,020	1,232	0,593	0,569
2,236	1,191	0,755	0,690
2,557	1,030	0,412	0,490
2,281	0,845	1,637	0,562
2,526	0,820	0,669	0,259
2,583	1,004	0,470	0,428
2,025	0,995	0,492	0,580
2,017	1,546	0,484	0,398
2,117	1,397	0,584	0,532
3,711	1,556	0,426	0,823
0,917	1,111	0,406	0,679
2,625	1,659	0,395	0,443
2,397	1,145	0,547	0,594
3,084	1,360	0,428	0,548
2,317	0,896	0,429	0,527
1,004	1,046	0,458	0,957
3,045	1,544	0,583	0,458
0,871	0,664	0,496	0,433
3,491	0,771	0,448	0,454
2,433	1,400	0,373	0,665
1,760	1,123	0,406	0,746
3,942	1,403	0,723	0,780
1,899	1,206	0,225	1,221
1,086	1,220	0,532	0,579
1,424	1,444	0,479	0,535
1,154	0,731	0,360	0,395
1,698	1,186	0,359	0,656
1,429	0,923	0,417	0,328
2,351	1,000	0,345	0,378
2,018	1,255	0,491	0,835
2,044	1,242	0,328	0,582
1,680	0,851	0,457	0,573
2,281	1,006	0,219	0,320
2,164	1,109	0,647	0,519
0,801	1,361	0,600	0,931
0,520	0,717	0,742	0,981
1,865	0,936	0,476	0,647
1,133	0,719	0,431	0,495
1,080	0,808	0,274	1,201
2,242	1,357	0,568	0,714
0,367	0,875	0,475	1,225
1,629	0,984	0,357	1,014
2,680	1,294	0,215	1,220
2,203	2,790	0,400	1,188
3,716	2,021	0,340	0,817
0,484	0,970	0,406	0,962

Figure 6b

	N	T_{CM}	T_{EM}	T_{TD}	α4+β1+	ICOS+	TIGIT+	HLA-DR+	PD-1+
	# of intact proviral sequence								
ART3			1		1				1
ART3			1		1			1	
ART3			1		1		1		
ART3			1						
ART3			1		1				
ART3			1		1				1
ART5			1		1				
ART6			1		1				1
ART6		1			1	1			1
ART6		1			1			1	1
ART6		1			1			1	
ART6		1			1				

Figure 6c

PID	mVLA4-	mVLA4+
	copies/10^6 CD4	
ART1	718	936
ART2	820	1570
ART3	1123	806
ART4	176	277
ART6	1420	2365
ART7	1835	2362
ART8	827	1203
ART9	197	433
ART10	338	765

Figure 6d

	mVLA4-	mVLA4+	
PID	IUPM		LOD
ART2	0,496	3,234	
ART3	0,248	0,279	
ART6	0,000	1,388	0,133
ART7	0,000	3,307	0,164
ART8	0,000	4,392	0,084
ART9	0,000	6,819	0,133

Supplementary 1

	Single-cell sorting efficiency	NFL HIV amplification efficiency	Sequencing fidelity
%	96,67	68,33	66,2

Supplementary 2a

	p24+						p24+					
	# of proviral sequence						# of proviral sequence					
	ART1	ART2	ART3	ART4	ART5	ART6	ART1	ART2	ART3	ART4	ART5	ART6
Unique	4	7	12	12	8	22	33	17	36	22	10	45
Clonal	48	48	38	29	44	36	35	37	17	29	14	31

Supplementary 2b

Proviral integrity	p24+ # of proviral sequence						p24- # of proviral sequence					
	ART1	ART2	ART3	ART4	ART5	ART6	ART1	ART2	ART3	ART4	ART5	ART6
Intact	0	0	6	0	1	5	0	0	0	0	7	0
Inversion	0	0	0	0	0	0	1	0	4	1	0	3
Hypermutations	0	0	0	0	0	0	9	0	5	4	0	8
Large deletion	0	36	3	10	5	10	55	54	42	34	12	56
Stop codons	0	9	2	0	1	31	0	0	1	0	1	7
Ψ defects	52	10	39	31	45	12	3	0	1	12	4	2

Supplementary 2c

	ART1 # of proviral sequence		ART2 # of proviral sequence		ART3 # of proviral sequence		ART4 # of proviral sequence		ART5 # of proviral sequence		ART6 # of proviral sequence	
Clone ID	p24+	p24-	p24+	p24-	p24+	p24-	p24+	p24-	p24+	p24-	p24+	p24-
I		2		37		2		2		2		2
II		2	33			2		3		2		2
III		4	4			3		4		2		4
IV		2	2			5		2	4	1		2
V		2	9			2		3		1		2
VI		2	1			2		3	3			15
VII		2			17		1	2				2
VIII		6			16	1	5	3	2		3	
IX		4			3		9	1	32	3	4	
X		2			2		3		1	5	4	1
XI		2					3	2	3		1	1
XII		3					5	3				2
XIII		2					4	2			4	1
XIV	4	1					1	1			12	
XV	44	1									3	
XVI											6	
Unique	4	31	6	17	12	36	10	20	7	8	22	42

Supplementary 2d

	p24+							p24-						
	# of proviral sequence							# of proviral sequence						
	Total	ART1	ART2	ART3	ART4	ART5	ART6	Total	ART1	ART2	ART3	ART4	ART5	ART6
MSD point mutation	16	0	0	1	5	3	7	4				3	1	
SL2 deletion	66	46	10	1	6	3	0	9	1		1	7		
Ψ deletion	107	6	0	37	20	39	5	9	2			2	3	2

Supplementary 5b

PID	**CD4**$^{+}$ T cells	**Memory CD4**$^{+}$ T cells
	**% of cells expressing VLA-4**	
ART1	17,55	35,92
ART2	28,08	29,38
ART3	33,88	39,78
ART4	35,5	49,36
ART6	17,96	32,41
ART7	10,26	24,95
ART8	30,37	38,99
ART9	17,47	44,6
ART10	22,28	34,3

Supplementary 5c

	% of memory CD4+ T cells VLA-4+ expressing						% of memory CD4+ T cells VLA-4- expressing						% of total CD4+ T cells expressing					
PID	PD-1	TIGIT	HLA-DR	ICOS	Tcm	Tem	PD-1	TIGIT	HLA-DR	ICOS	Tcm	Tem	PD-1	TIGIT	HLA-DR	ICOS	Tcm	Tem
ART1	24,19	38,17	8,38	29,08	59,55	27,32	13,6	11,1	17	16,4	24,4	5,7	6,99	10,96	10,31	20,08	17,04	7,73
ART2	37,44	38,89	14,22	39,61	42,1	49,39	23,9	12,4	30	37	45,7	29	23,21	24,87	13,63	38,35	27,41	32,39
ART3	48,83	20,34	2,4	16,64	22,75	62,58	13,6	5,3	17,2	27,2	52	14,6	32,72	13,4	3,38	20,1	15,07	41,6
ART4	36,11	32,98	3,92	26,02	54,89	36,66	15,9	6,1	21	19,2	32,8	8,9	16,25	15	5,11	22,4	25,14	16,2
ART5	41,75	41,1	6,24	42,2	48,26	36,43	19,7	6,9	21,9	27,7	28,3	8,3	16,3	16,19	6,69	33,45	18,94	14,45
ART6	17,3	32,21	8,99	33,04	55,74	10,68	8,4	8,8	21,4	27,2	33,7	7	8,23	15,39	8,86	30	25,95	4,88
